# A prescription support-tool for chronic management of oral antithrombotic combinations in adults based on a systematic review of international guidelines

**DOI:** 10.1371/journal.pone.0211695

**Published:** 2019-02-14

**Authors:** Lorene Zerah, René-Sosata Bun, Sylvie Guillo, Jean-Philippe Collet, Dominique Bonnet-Zamponi, Florence Tubach

**Affiliations:** 1 Sorbonne Université, INSERM, Institut Pierre Louis d’Epidémiologie et de Santé Publique, AP-HP, Hôpital Pitié-Salpêtrière, Département Biostatistique Santé Publique et Information Médicale, Unité de Recherche Clinique PSL-CFX, Centre de Pharmacoépidémiologie (Cephepi), Paris, France; 2 Sorbonne Université, INSERM, AP-HP, Hôpital Pitié-Salpêtrière, Département de Cardiologie, Paris, France; 3 Observatoire du Médicament des Dispositifs Médicaux et de l’Innovation Thérapeutique Ile de France (OMEDIT), Paris, France; Universite de Bretagne Occidentale, FRANCE

## Abstract

**Background:**

Oral antithrombotic (AT) drugs, which include antiplatelet and anticoagulant therapies, are widely implicated in serious preventable bleeding events. Avoiding inappropriate oral AT combinations is a major concern. Numerous practical guidelines have been released; a document to enhance prescriptions of oral AT combinations for adults would be of great help.

**Objective:**

To synthesize guidelines on the prescription of oral AT combinations in adults and to create a prescription support-tool for clinicians about chronic management (≥ one month) of oral AT combinations.

**Methods:**

A systematic review of guidelines published between January 2012 and April 2017, in English or in French, from Trip database, Guideline International Network and PubMed, dealing with the prescription of oral ATs in adults was conducted. In-hospital management of ATs, bridging therapy and switches of ATs were not considered. Some specific topics requiring specialized follow-up (cancer, auto-immune disease, haemophilia, HIV, paediatrics and pregnancy) were excluded. Last update was made in November 2018.

**Results:**

A total of 885 guidelines were identified and 70 met the eligibility criteria. A prescription support-tool summarizing medical conditions requiring chronic management of oral AT combinations in adults with drug types, dosage and duration, on a double-sided page, was provided and tested by an external committee of physicians. The lack of specific guidelines for old people (age 75 years and older) is questioned considering the specific vulnerability of this age group to serious bleedings.

**Conclusions:**

Recommendations on prescriptions about chronic management of oral AT combinations in adults were mainly consensual but dispersed in numerous guidelines according to the medical indication. We provide a prescription support-tool for clinicians. Further studies are needed to assess the impact of this tool on appropriate prescribing and the prevention of serious adverse drug events.

## Introduction

Combinations of oral antithrombotic (AT) drugs, which include antiplatelet (AP) and anticoagulant (AC) therapies, are increasingly being prescribed in relation to the increase in prevalence of cardiovascular diseases, multimorbidity (commonly defined as the presence of 2 or more chronic medical conditions in an individual) and medical progress.[1]

Combinations of ATs have demonstrated their benefit in various medical neuro-cardiovascular conditions, but they increase widely the risk of severe bleeding.[2,3] For example, Hansen et al. reported a 3.1-fold higher risk for fatal and non-fatal bleedings with dual warfarin and clopidogrel therapy and a 3.7-fold higher risk with triple therapy (warfarin, aspirin and clopidogrel) than warfarin monotherapy in patients with non-valvular atrial fibrillation (NV-AF).[3] ATs are already implicated, alone or in combination, in almost 25% of adverse drug events (ADEs) leading to emergency department visits in the general population (almost 50% of ADEs in patients age 80 years and older), with subsequent hospitalization in almost half of the cases.[2] They are also implicated in more than half of suspected fatal ADEs.[4] During the last decade, the proportion of emergency department visits related to ADEs involving ACs has increased by 57%, along with a 38% increase in ACs use.[2,5] Some of these ADEs are not preventable (related to patient idiosyncrasy or unforeseeable accidents). However, a recent review demonstrated that AT is one of the therapeutic classes the most implicated in preventable ADEs leading to hospitalization.[6]

Developing efficient risk minimization actions is necessary to improve the benefit/risk ratio of ATs. Improving their prescriptions by avoiding their inappropriate combinations (in terms of indication, dosage, type of drugs combined and duration of prescription) is a major concern. In a Canadian primary care cohort, approximately 15% of patients who were prescribed ATs had inappropriate dual or triple oral AT therapies (type of drugs combined only),[7] which suggests an important room for improvement for prescription of oral AT combinations. Actually, most clinical practice guidelines focus on a single disease and applying single-disease guidelines for multimorbidity increases the risk of inappropriate prescriptions (among other things inappropriate combinations).[8] In a clinician perspective, an easily readable access to the last and accurate recommendation that is relevant for his/her patient’s clinical situation would be very valuable and of great interest.

Our main objective was to synthesize, in a prescription support-tool, the guidelines for chronic management (at least 1 month) of oral AT combinations in adults (thus excluding in-hospital management of ATs, bridging therapy and switches of ATs). Our secondary objective was to determine whether recommendations for oral AT combinations differed with higher age, older adults (age 75 years and older) having increased risk for serious bleeding due to comorbidities and age-related physiologic changes.[9]

## Methods

Our review was developed following the PRISMA statement for systematic reviews[10] **(**S1 Appendix**).** We did not declare a protocol for our systematic review of guidelines.

### Inclusion and non-inclusion criteria

We included all guidelines published in English or French, between January 1, 2012 and April 04, 2017, dealing with the use of oral ATs (APs and ACs) for NV-AF, coronary artery disease, ischemic stroke, valvular heart disease, peripheral artery disease and venous thromboembolism in adults (S2 Appendix). Last update was made in November 2018. These pathologies were selected because most prescriptions of ATs are related to neuro-cardiovascular diseases[1] and because we would provide a synthesis relevant for clinicians in charge of the follow-up of patients with oral AT combinations. In-hospital management of ATs, bridging therapy and switches of ATs were not considered. We excluded guidelines pertaining to rare conditions sometimes leading to AT combinations and requiring specialized care: active cancer, autoimmune diseases, haemophilia, HIV, paediatrics and pregnancy. The target clinicians for the prescription support-tool are those implicated in the follow-up of adults with multimorbidity, namely general practitioners, geriatricians, internists, and cardiologists.

### Search strategy, data extraction and quality assessment

Both, the search and guidelines selection, were conducted by two independent readers (LZ, internist and geriatrician; and RSB, pharmacist) using guideline-specific websites (Trip database, Guideline International Network) and PubMed. The search strategy (eligibility criteria, data sources, selection process and search terms) is detailed in S2 Appendix**.**

For the two major indications for chronic management of oral AT combinations (i.e., NV-AF and coronary artery disease), two independent readers (LZ and DBZ, internist and geriatrician) extracted data and double-entered it into a table organized by drugs and medical conditions. Because the agreement between readers was 100%, only LZ performed data extraction for other medical conditions (decision made *a priori*). If discordances between guidelines were collected about AT combinations, in terms of number of AT drugs that had to be used (single, dual or triple AT therapy) or in terms of duration and dosage, it was decided a priori that the final choice of the retained recommendation would be performed by a scientific committee including all authors (LZ; RSB; DBZ; FT, public health and epidemiology; JPC, cardiologist; SG, epidemiologist expert in systematic reviews) considering the level of evidence of recommendations, the number of similar recommendations and their publication date. Specific recommendations about the elderly (defined as patients aged 75 years old or older) were also systematically collected.

### Data synthesis and analysis: Design of the prescription support-tool and test by an external committee of physicians

From this exhaustive table of recommendations, a more operational synthesis was created summarizing recommended indications and duration for chronic management of oral AT combinations (prescription support-tool). An external committee of physicians, representing the target of the tool, was set up to assess the layout and the usefulness of this prescription support-tool from May to June 2017. The external committee involved 7 general practitioners (2 affiliated with a hospital), 3 geriatricians (one practicing in a hospital, one in outpatient setting and one in a nursing home), one general cardiologist (practicing in outpatient setting), one internist (practicing in a hospital), one emergency clinician and one biologist specialized in haemostasis (practicing in a hospital). Eleven of these physicians practiced in Paris and 3 in rural areas. They were asked about the approximate proportion of patients with oral AT combinations in their practice (< 5%, 5–10%, 10–20% or > 20%), whether they feel comfortable or not with management of oral AT prescriptions (totally, partially, rarely, never) and whether they know where to find the most recent guidelines on oral AT prescriptions. Finally, we asked them to rate on a scale from 0 and 10 the usefulness of the prescription support-tool, how much they would be willing to use this prescription support-tool in their practice and if they would recommend its use.

## Results

**Fig 1:**
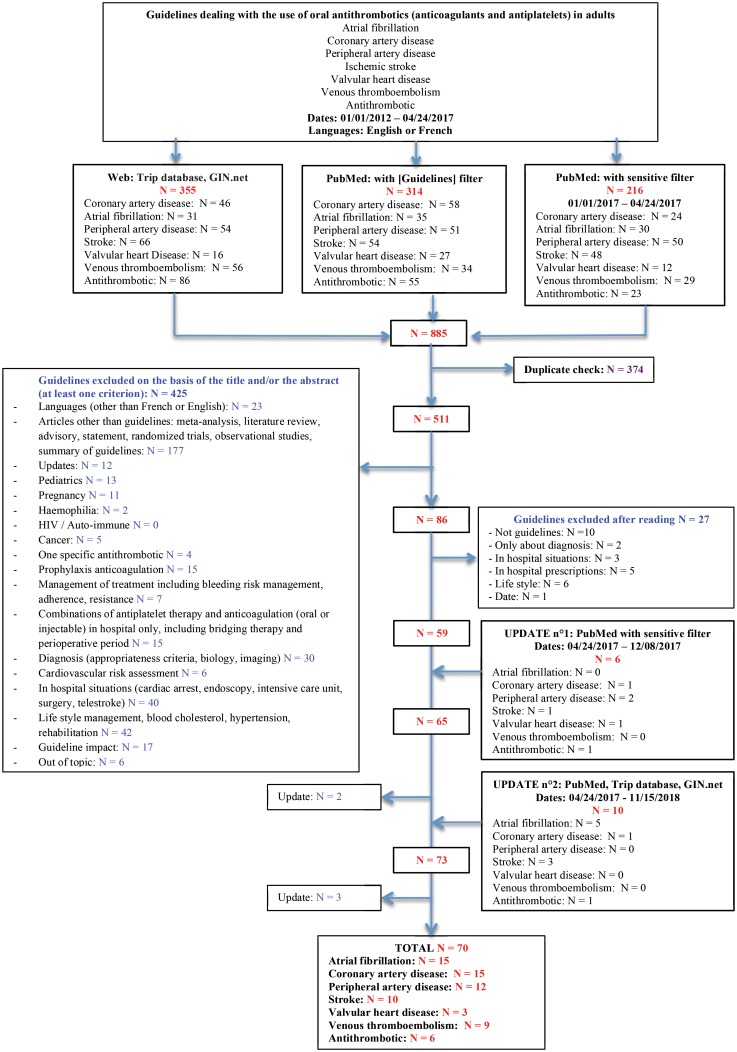
PRISMA chart.

A total of 885 guidelines were found according to the algorithm search; 70 met the eligibility criteria (Fig 1**)** and covering the following topics: atrial fibrillation (n = 15), coronary artery disease (n = 15), peripheral artery disease (n = 12), stroke (n = 10), valvular heart disease (n = 3), venous thromboembolism (n = 9) and antithrombotics (n = 6).[11–80]

**Fig 2:**
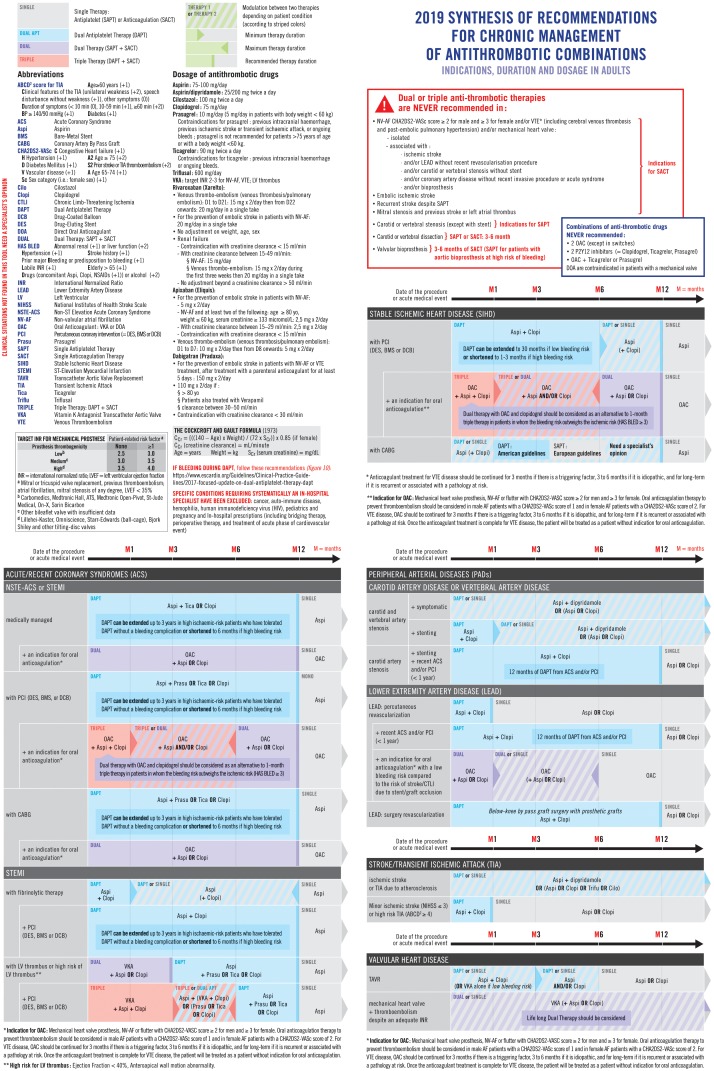
2019 synthesis of recommendations for combining antithrombotic drugs. Indication, duration and dosage in adult patients.

All guidelines were in English; the average time delay between each update of guidelines was 5 years. However, this delay was shorter in guidelines released by the European Society of Cardiology for which several clinical situations overlapped in guidelines issued less than 1 year apart.[22,80] Despite being released from various geographical areas including Africa (n = 1), Asia (n = 14), Brazil (n = 1), Canada (n = 10), Europe (n = 13), New Zealand-Australia (n = 4), United Kingdom (n = 9) and the United States (n = 18), the numerous recommendations on oral AT combinations duration, drugs and dosage were mostly consensual. Only one clinical situation was found for which guidelines were discordant on oral AT combinations that had to be used: stable ischemic heart disease with a coronary artery bypass graft. Two guidelines made recommendations about this specific situation. American guidelines (2016)[11] recommend a dual antiplatelet therapy for 12 months with a grade IIB (“it may be reasonable”); the European guidelines (2017)[80] recommend no dual antiplatelet therapy (no grade).[81] Considering that there were only two guidelines published in 2016 and 2017 with a low level of evidence, the scientific committee did not choose between these two guidelines and integrated these two strategies in the tool (Fig 2**)**, recommending a specialist’s opinion. For this specific situation, the treatment is always initiated at hospital. Therefore, out-of-hospital physicians will be able to continue what the specialist has started.

Indications, type of drugs combined, dosage of drugs, duration of prescription and grade of recommendations (American and European guidelines) are outlined in S1 Table and synthesized in Fig 2 (prescription support-tool). All clinical situations synthesized in our tool were consensual, but not always recommended with a grade I (“Evidence and/or general agreement that a given treatment is indicated, beneficial, useful, effective” [81]). In particular, indications of dual or triple therapy were recommended most of the time with a grade II (“Conflicting evidence and/or a divergence of opinion about the usefulness/efficacy of the given treatment: should or may be considered”[81]) (S1 Table**)**. A consistent message of all guidelines is that the duration of combinations of AT is flexible and may be adapted (prolonged or shortened) according to patient-specific risks of ischemia and bleeding[81] assessed respectively by the CHA_2_DS_2_-VASc (Congestive heart failure, Hypertension, Age ≥ 75 years, Diabetes mellitus, prior stroke or thromboembolism, Vascular disease, Age 65–74 years, Sex) and the HAS BLED scores (Hypertension, Abdnormal renal and liver function, Stroke, Bleeding, Labile INR, Elderly, Drugs or alcohol).[82,83] We also included in the prescription support-tool the contraindicated oral AT combinations (combinations of oral anticoagulant (OAC), combinations of P2Y12 inhibitors or the combination of one OAC with one potent P2Y12 inhibitor, namely ticagrelor or prasugrel), the clinical situations where oral AT combinations were never required and contraindications of ATs, as specified into the guidelines. None recommendation was found for some associations of diseases (for example: transcatheter aortic valve replacement and pulmonary embolism or NV-AF) and thus were not described in the prescription support-tool. Indeed, our aim was to synthesize available guidelines and not to provide new guidelines in clinical situations not covered. For these situations, a specialist advice is recommended and this is stated in the tool: “clinical situations not found in this tool need a specialist’s opinion”. We developed a user guide to facilitate the initial use of the prescription support-tool **(**S3 Appendix**).**

### Focus on recommendations for older people

Only few guidelines (n = 13) specified elderly as a subgroup of interest.[18,22,23,25,28,30,34,36,38,39,41,63,67] Only 4 of these guidelines defined elderly: ≥ 75 years old in Malaysian[23,25] and American guidelines,[30] ≥ 65 years old in one European guideline.[28] Nevertheless, no specific guidance was provided but rather caution: prescribers should be more careful with this population **(**S2 Table**).** The only restriction pertained the use of prasugrel in patients ≥ 75 years where a lower dose regimen of 5 mg/day should be used if treatment was deemed necessary.[22,80]

### Test of the tool by the external committee of physicians

Internists and cardiologists estimated that 10 to 20% of their patients were treated with oral AT combinations; general practitioners estimated that less than 5% of their patients were treated with oral AT combinations, the other specialties estimated that 5 to 10% of their patients were treated with oral AT combinations. Most physicians in the external committee (10/14, 71.4%) were not at all or not really comfortable with the chronic management of oral AT combinations and reported being scared about these prescriptions (11/14, 78.6% totally or moderately agreed with “the prescription of oral AT combinations scares me”). Few declared to know perfectly where to find the most recent guidelines about chronic management of oral AT combinations (3/14, 21.4%). Overall, the tool was found useful for clinical practice (mean score 9.1/10). Many physicians were ready to use it (mean score 9.4/10), and to recommend its use (mean score 9.2/10). Criticisms on the layout and notably on the maximum and minimum recommended durations were made leading to a reshape version by a graphic designer (Fig 2**)**. Some physicians, especially those working in the outpatient setting, expressed the wish to also have an e-tool and the possibility to integrate the recommendations into their prescription support software.

## Discussion

We propose a prescription support-tool, on a double-sided page, synthesizing all national and international guidelines about chronic management (at least one month) of oral AT combinations (drugs, dosages and duration) in adults (Fig 2**)**. Synthesis figures were already released in some guidelines, but none encompassed all the clinical situations either isolated or associated requiring oral AT combinations. However, to gather all recommendations into one document may be the key to enhance accurate prescription of oral ATs for patients with multiple chronic conditions, more than half of whom have cardiovascular diseases.[84]

The systematic review that led to the development of this tool demonstrated the multiplicity of guidelines dealing with oral AT combinations (n = 70 within 5 years). This was accounted for by the wealth of clinical trials on this topic[22,27,77,80,81] and the many overlapping situations between guidelines. This highlights how difficult it can be for a clinician to know exactly where to find the most up-to-date guideline that is relevant for his/her patient’s clinical situation, especially if the patient had several comorbidities. Furthermore, the short time delay between updates[85,86] with sometimes inconsistencies on the same situation, make things even more complex. For example, the PREdicting bleeding Complications In patients undergoing Stent implantation and subsEquent Dual Anti Platelet Therapy (PRECISE-DAPT) score released in the 2017 DAPT update of the European Society of Cardiology[80] to risk stratify patients at high bleeding risk and not deserving long-term DAPT was not mentioned in the 2017 European guidelines.[22] Given a class IIb with a level of evidence A, we chose not to integrate this score in our clinical tool yet. This decision will be revaluated with the publication of the future guidelines.

In total, the dispersal of information into numerous guidelines and the frequent updates could prevent clinicians’ easy access (especially those with general practice) to the most current and consensual recommendations and could explain a part of inappropriate prescribing. It could explain also the feeling of discomfort and the fear of prescribing AT combinations expressed by many of the reading-committee physicians. It reinforced the relevance to propose this prescription support-tool synthesizing all the recommendations in one document. From this tool, clinicians will access easily to the recommended prescription of AT according to their patients’ clinical situations. Our goal is to reduce inappropriate prescription of oral AT combinations, not to recommend one AT molecule over another one, especially because of different practices and permissions depending on geographical locations.

Older people are at the greatest risk for AT-related severe bleeding events[9] for two main reasons. First, the prevalence of multimorbidity increases with age,[87] thereby enhancing the risk for ADEs and drug–drug and drug–disease interactions. Second, ageing is an independent risk factor for bleeding, and ATs prescription further and variously amplifies this age-related bleeding risk.[9] However, we found no guideline on AT combinations dedicated to older adults. Two major reasons could explain the paradoxical lack of specific and detailed guidelines for older people. First, older people are underrepresented in trials exploring the benefit of AT drugs. For example, more than one third of patients admitted to hospital with acute coronary syndrome (ACS) and two thirds dying from ACS are > 75 years old, but less than 7% of patients in ACS trials are reported to be > 75 years old.[88] Second, the definition of older adults is large and encompasses old, old-old and oldest-old individuals with different prognoses.[89,90] Therefore, there is an urgent need to define/refine AT therapy for the oldest groups to maximize benefits and minimize risks. To avoid older people being denied AT drugs because of unjustified concerns or conversely being inappropriately overtreated, the European Society of Cardiology Working Group on Thrombosis, in 2015, proposed a patient-oriented consensus document focused on age-specific risks and benefits of AT drugs tested in phase III trials.[91] However, studies of the benefit/risk ratio of AT combinations in real life in the different categories of older people are needed to support evidence-based data. While awaiting the results of these studies, the relevance of consensus-based recommendations of AT combinations for the oldest-old individuals (≥ 85 years old) still remains.

### Strengths and limits

This is the first systematic review synthesizing all existing national and international guidelines on the prescription of chronic management of oral AT combinations in adults. From this synthesis, we developed a prescription support-tool **(**Fig 2) to improve prescriptions in this field, which is of major concern, especially by avoiding inappropriate oral AT combinations.

Our review had some limitations. First, we excluded from this study recommendations for injectable ACs (long-term or bridging therapy with a short period of AT combinations), although these situations were also AT combinations at risk of ADEs.[92,93] Long-term injectable AC therapy was excluded because it was usually prescribed for a thromboembolism episode in the context of active cancer[70,72,79,94,95] and therefore the situation should be resolved individually with a cancer specialist. The usually transitional nature of bridging therapy led us to exclude these situations.[92,93] Second, scant evidence exists for the compilation of existing guidelines, and the generation of clinical practice guidance upon guidelines assimilation. The most widely accepted method to assess clinical guidelines is the AGREE (Appraisal of Guidelines Research and Evaluation) instrument (revised version AGREE II published in 2009[96]). However, the overall assessments of AGREE II are highly subjective and a standardized approach to reaching these assessments is lacking.[96] In our case, our main objective was not to choose the “best” guideline but to synthesize them into one simple tool, accounting for multimorbidity. Finally, we included only guidelines and not literature reviews, meta-analyses or results of randomized trials because our aim was not to perform new recommendations. Other studies testing antithrombotic strategies are currently ongoing and could change the recommendations[81], which is why we plan to make updates every 6 months thanks to a dedicated team, and to make them available through a free interactive web version of this tool (work on process).

## Conclusion

This is the first prescription support-tool synthesizing national and international guidelines on chronic management of oral AT combinations (drugs, dosages and duration) in adults. Further studies are needed to demonstrate the impact of this tool on AT inappropriate prescribing and on clinical outcomes. The review used to design this tool underlined also the lack of accurate guidelines regarding dual and triple AT therapies for the older people, although they are most at risk for severe bleeding events. Further research must focus on this population, especially the oldest-old individuals and those with frailty, to refine the recommendations.

## Supporting information

S1 Appendix PRISMA checklist. (DOCX)

S2 Appendix Search strategy (eligibility criteria, information sources, selection process). (DOCX)

S3 Appendix Prescription support-tool’s user guide. (PDF)

S1 Table Synthesis of recommendations from all selected guidelines dealing with the use of oral antithrombotic (AT) drugs: Indications, duration and dosage in adults. (DOCX)

S2 Table Specificities from guidelines about older people. (DOCX)
