# Supplementary material for: A prescription support-tool for chronic management of oral antithrombotic combinations in adults based on a systematic review of international guidelines
Source: PLoS One. 2019 Feb 14;14(2):e0211695. doi: 10.1371/journal.pone.0211695 (PMC6375571; doi:10.1371/journal.pone.0211695)
Supplement: S1 Appendix — (DOCX) [file pone.0211695.s001.docx]

**S1 Appendix: Prisma checklist**

| **Section/topic** | **Item No** | **Checklist item** | **Reported on page No** |
| --- | --- | --- | --- |
| **Title** |  |  |  |
| Title | **1** | Identify the report as a systematic review, meta-analysis, or both | **Page 1, title “a systematic review”** |
| **Abstract** |  |  |  |
| Structured summary | **2** | Provide a structured summary including, as applicable, background, objectives, data sources, study eligibility criteria, participants, interventions, study appraisal and synthesis methods, results, limitations, conclusions and implications of key findings, systematic review registration number | **Page 2, abstract** |
| **Introduction** |  |  |  |
| Rationale | **3** | Describe the rationale for the review in the context of what is already known | **Pages 3-4**  **Lines 48-76** |
| Objectives | **4** | Provide an explicit statement of questions being addressed with reference to participants, interventions, comparisons, outcomes, and study design (PICOS) | **Page 4**  **Lines 77-82** |
| **Methods** |  |  |  |
| Protocol and registration | **5** | Indicate if a review protocol exists, if and where it can be accessed (such as web address), and, if available, provide registration information including registration number | **Page 4**  **Line 85-86** |
| Eligibility criteria | **6** | Specify study characteristics (such as PICOS, length of follow-up) and report characteristics (such as years considered, language, publication status) used as criteria for eligibility, giving rationale | **Pages 4-5**  **Lines 88-100**  **S2 Appendix** |
| Information sources | **7** | Describe all information sources (such as databases with dates of coverage, contact with study authors to identify additional studies) in the search and date last searched | **Page 5**  **Lines 102-105**  **S2 Appendix** |
| Search | **8** | Present full electronic search strategy for at least one database, including any limits used, such that it could be repeated | **S2 Appendix** |
| Study selection | **9** | State the process for selecting studies (that is, screening, eligibility, included in systematic review, and, if applicable, included in the meta-analysis) | **Page 5**  **Lines 102-105**  **Figure 1**  **S2 Appendix** |
| Data collection process | **10** | Describe method of data extraction from reports (such as piloted forms, independently, in duplicate) and any processes for obtaining and confirming data from investigators | **Page 5**  **Lines 106-118**  **S2 Appendix** |
| Data items | **11** | List and define all variables for which data were sought (such as PICOS, funding sources) and any assumptions and simplifications made | **Pages 4-5**  **Lines 88-100** |
| Risk of bias in individual studies | **12** | Describe methods used for assessing risk of bias of individual studies (including specification of whether this was done at the study or outcome level), and how this information is to be used in any data synthesis | **Not adapted to our review (only guidelines)** |
| Summary measures | **13** | State the principal summary measures (such as risk ratio, difference in means). | **Not adapted to our review (only guidelines)** |
| Synthesis of results | **14** | Describe the methods of handling data and combining results of studies, if done, including measures of consistency (such as I2 statistic) for each meta-analysis | **Page 5**  **Lines 106-118**  **Pages 6:**  **Lines 121-123** |
| Risk of bias across studies | **15** | Specify any assessment of risk of bias that may affect the cumulative evidence (such as publication bias, selective reporting within studies) | **Not adapted to our review (only guidelines)** |
| Additional analyses | **16** | Describe methods of additional analyses (such as sensitivity or subgroup analyses, meta-regression), if done, indicating which were pre-specified | **Page 5:**  **Lines 117-118**  **Page 6**  **Lines 123-136** |
| **Results** |  |  |  |
| Study selection | **17** | Give numbers of studies screened, assessed for eligibility, and included in the review, with reasons for exclusions at each stage, ideally with a flow diagram | **Page 6**  **Lines 139-142**  **Figure 1** |
| Study characteristics | **18** | For each study, present characteristics for which data were extracted (such as study size, PICOS, follow-up period) and provide the citations | **Not adapted to our review (only guidelines)** |
| Risk of bias within studies | **19** | Present data on risk of bias of each study and, if available, any outcome-level assessment (see item 12). | **Not adapted to our review (only guidelines)** |
| Results of individual studies | **20** | For all outcomes considered (benefits or harms), present for each study (a) simple summary data for each intervention group and (b) effect estimates and confidence intervals, ideally with a forest plot | **Not adapted to our review (only guidelines)** |
| Synthesis of results | **21** | Present results of each meta-analysis done, including confidence intervals and measures of consistency | **Pages 7-8**  **Lines 144-185**  **Figure 2**  **S1 Table (with grade of recommendations for European and American guidelines)**  **S3 Appendix** |
| Risk of bias across studies | **22** | Present results of any assessment of risk of bias across studies (see item 15) | **Not adapted to our review (only guidelines)** |
| Additional analysis | **23** | Give results of additional analyses, if done (such as sensitivity or subgroup analyses, meta-regression)  (see item 16) | **Pages 8-9**  **Lines 188-211** |
| **Discussion** |  |  |  |
| Summary of evidence | **24** | Summarize the main findings including the strength of evidence for each main outcome; consider their relevance to key groups (such as health care providers, users, and policy makers) | **Pages 9-11**  **Lines 214 – 266** |
| Limitations | **25** | Discuss limitations at study and outcome level (such as risk of bias), and at review level (such as incomplete retrieval of identified research, reporting bias) | **Pages 11-12**  **Lines 267-291** |
| Conclusions | **26** | Provide a general interpretation of the results in the context of other evidence, and implications for future research | **Page 13**  **Lines 293-300** |
| **Funding** |  |  |  |
| Funding | 27 | Describe sources of funding for the systematic review and other support (such as supply of data) and role of funders for the systematic review | **Not described (as requested by the journal)** |
