# Supplementary material for: A prescription support-tool for chronic management of oral antithrombotic combinations in adults based on a systematic review of international guidelines
Source: PLoS One. 2019 Feb 14;14(2):e0211695. doi: 10.1371/journal.pone.0211695 (PMC6375571; doi:10.1371/journal.pone.0211695)
Supplement: S2 Appendix — (DOCX) [file pone.0211695.s002.docx]

**S2 Appendix: Search strategy (eligibility criteria, information sources, selection process)**

**Information sources and selection process:** The research involved:

- Tridatabase: [www.tripdatabase.com](http://www.tripdatabase.com)
- GIN (Guidelines International Network): <http://www.g-i-n.net/>
- PubMed: [www.ncbi.nlm.nih.gov/pubmed](http://www.ncbi.nlm.nih.gov/pubmed)

**Inclusion criteria:**

- Guidelines on pathologies leading to the prescription of oral antithrombotics in adults: atrial fibrillation, coronary artery disease, peripheral artery disease, ischemic stroke, valvular heart disease, venous thromboembolism disease
- Guidelines on oral antithrombotics (anticoagulants and antiplatelets) in adults prescribed for the previous pathologies
- Period: published from January 1, 2012 to April 24, 2017 (updates in December 2017 and November 2018)
- Languages: French and English
- Population: adults

**Exclusion criteria:**

- Articles other than guidelines: meta-analysis, literature review, advisory, statement, randomized trials, observational studies
- Updates: only the last guideline on the same subject and from the same society is retained
- Populations and diseases:
  - Paediatrics
  - Pregnancy
  - Auto-immune disease, haemophilia, HIV, cancer
- Guidelines on antithrombotics that focus only on:
  - One specific antithrombotic
  - In hospital situations, including bridging therapy and perioperative period
  - Prophylaxis anticoagulation
  - Management of treatment, including bleeding risk management, adherence, resistance
- Guidelines on selected pathologies that focus only on:
  - Diagnosis, cardiovascular risk assessment
  - Life style management, blood cholesterol, hypertension, rehabilitation
  - In hospital situations (cardiac arrest, endoscopy, ICU, surgery, telestroke)
  - Guideline impact

**Updates:**

- A first research was carried out from January 1, 2012 to April 24, 2017.
- An update was made on December 8, 2017 (04/24/2017 – 12/08/2017) using PubMed with sensitive filter to obtain guidelines not yet classified as guidelines.
- A second update (using PubMed, Trip database and GIN) was made on November 15, 2018 (04/24/2017 - 11/15/2018).

**The search terms, based on inclusion criteria, are described above.**

| **Search Algorithm: For each pathology and antithrombotic drug, we have realized this search algorithm that is described above (the dotted lines are related to the “search terms”)** | |
| --- | --- |
|  | **WEB**   - **Tripdatabase:** (Title: "…………") Filters: Guideline; Publication date from 01/01/2012 – 04/24/2017 (Update: 04/24/2017 - 11/15/2018) - **G-I-N.net:** (Title: "…………") Filters: Guideline; Publication date from 01/01/2012 – 04/24/2017 (Update: 04/24/2017 - 11/15/2018)   **PUBMED**   - **Pubmed with ‘Guideline’ filter**   Search "…………"[title] Filters: Guideline; Publication date from January 1, 2012 to April 24, 2017; French; English (Update: 04/24/2017 - 11/15/2018)   - **Pubmed with “sensitive” filter**   Search “…………”[Title] AND (Guideline[Title] OR Recommendation[Title] OR Update[Title]) Filters: Publication date from January 1, 2017 to April 24, 2017; French; English (Update: 04/24/2017 - 12/08/2017 and 12/08/2017 - 11/15/2018) |
| **Search terms** | |
| **Coronary Artery**  **Disease** | “Coronary artery disease”  “Coronary syndromes”  “Myocardial infarction”  “Ischemic heart disease” |
| **Atrial Fibrillation** | “Atrial fibrillation”  “Supraventricular tachycardia” |
| **Peripheral Artery Disease**  **and**  **Cardiovascular disease** | “Atherosclerotic occlusive disease”  “Extracranial carotid”  “Vertebral artery disease”  “Peripheral artery disease”  “Cardiovascular risk”  “Cardiovascular disease” |
| **Stroke** | “Stroke”  “Transient ischemic attack”  “Cerebral venous sinus thrombosis” |
| **Valvular Heart**  **Disease** | “Valvular disease”  “Valvular heart disease”  “Aortic valve”  “Mitral valve” |
| **Venous thromboembolism disease** | “Venous thromboembolism disease”  “Pulmonary embolism”  “Deep vein thrombosis” |
| **Antithrombotic** | “Antithrombotic”  “Antithrombins” |
| **Anticoagulant** | “Anticoagulant”  “Anticoagulation” |
| **Antiplatelet** | “Antiplatelet”  “Platelet aggregation inhibitors” |
