# Supplementary material for: A prescription support-tool for chronic management of oral antithrombotic combinations in adults based on a systematic review of international guidelines
Source: PLoS One. 2019 Feb 14;14(2):e0211695. doi: 10.1371/journal.pone.0211695 (PMC6375571; doi:10.1371/journal.pone.0211695)
Supplement: S3 Appendix — (PDF) [file pone.0211695.s003.pdf]

How to use the prescription support tool

General presentation of the prescription support tool

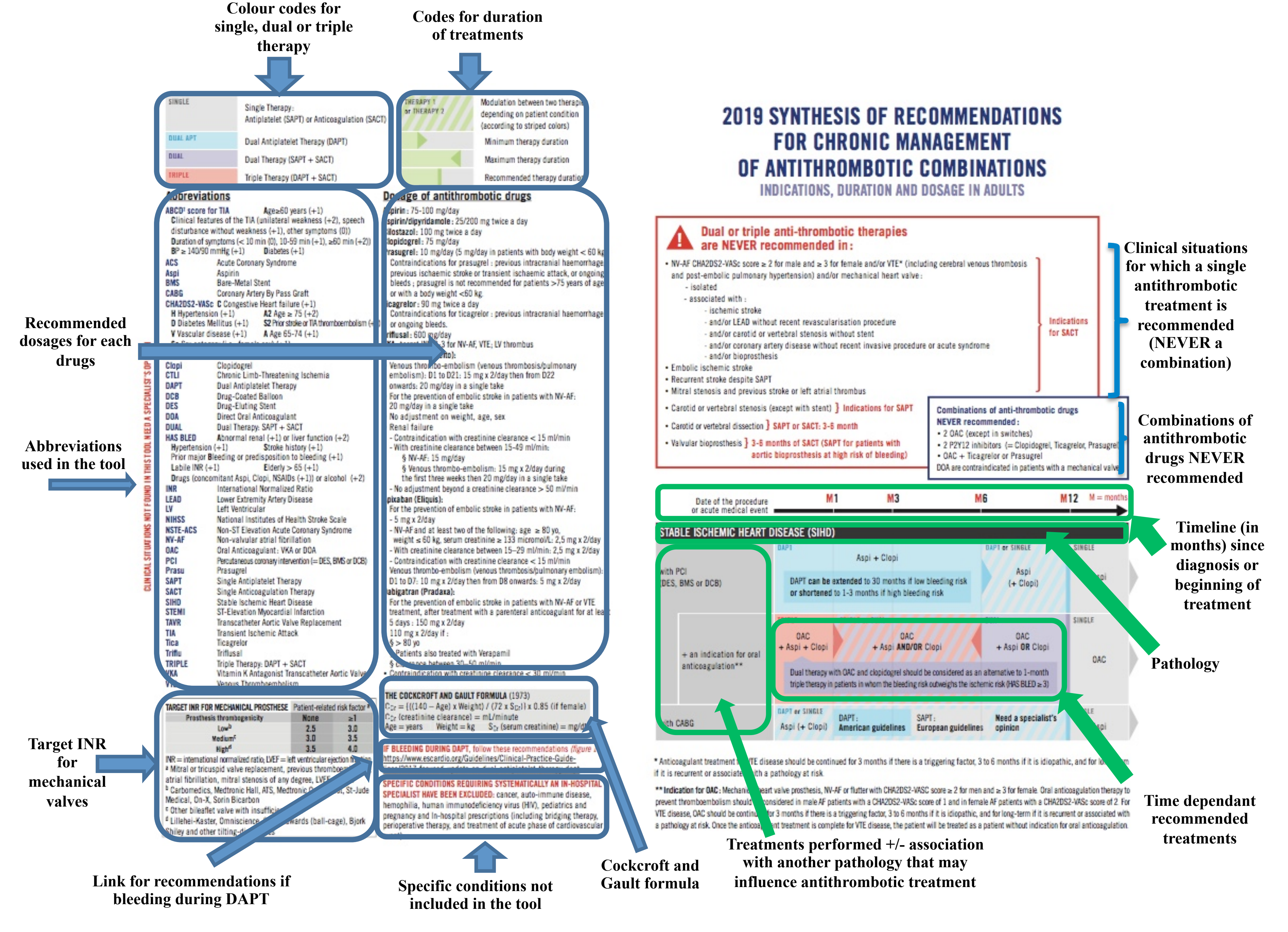
Colour codes for single, dual or triple therapy
Codes for duration of treatments
Recommended dosages for each drugs
Abbreviations used in the tool
Target INR for mechanical valves
Link for recommendations if bleeding during DAPT
Specific conditions not included in the tool
Cockcroft and Gault formula
Treatments performed +/- association with another pathology that may influence antithrombotic treatment
Time dependant recommended treatments
Pathology
Timeline (in months) since diagnosis or beginning of treatment
Combinations of antithrombotic drugs NEVER recommended
Clinical situations for which a single antithrombotic treatment is recommended (NEVER a combination)
2019 SYNTHESIS OF RECOMMENDATIONS FOR CHRONIC MANAGEMENT OF ANTITHROMBOTIC COMBINATIONS
INDICATIONS, DURATION AND DOSAGE IN ADULTS
Dual or triple anti-thrombotic therapies are NEVER recommended in:
Abbreviations
Dosage of antithrombotic drugs
THE COCKCROFT AND GAULT FORMULA (1973)
IF BLEEDING DURING DAPT, follow these recommendations
SPECIFIC CONDITIONS REQUIRING SYSTEMATICALLY AN IN-HOSPITAL SPECIALIST HAVE BEEN EXCLUDED
TARGET INR FOR MECHANICAL PROSTHESE
STABLE ISCHEMIC HEART DISEASE (SIHD)
M1
M3
M6
M12

In practice

Example 1: one cardiovascular disease

- At your medical consultation, you meet Mr R, 85 years old (weight: 81 kg, body mass index: 24 kg/m^2).
- Medical history: arterial hypertension and Parkinson disease
- He had surgery 8 months ago for an aortic stenosis: transcatheter aortic valve replacement (TAVR)
- Which antithrombotic therapy is recommended in this clinical situation?

1- Locate in the chapter headings of the tool, the cardiovascular disease of your patient

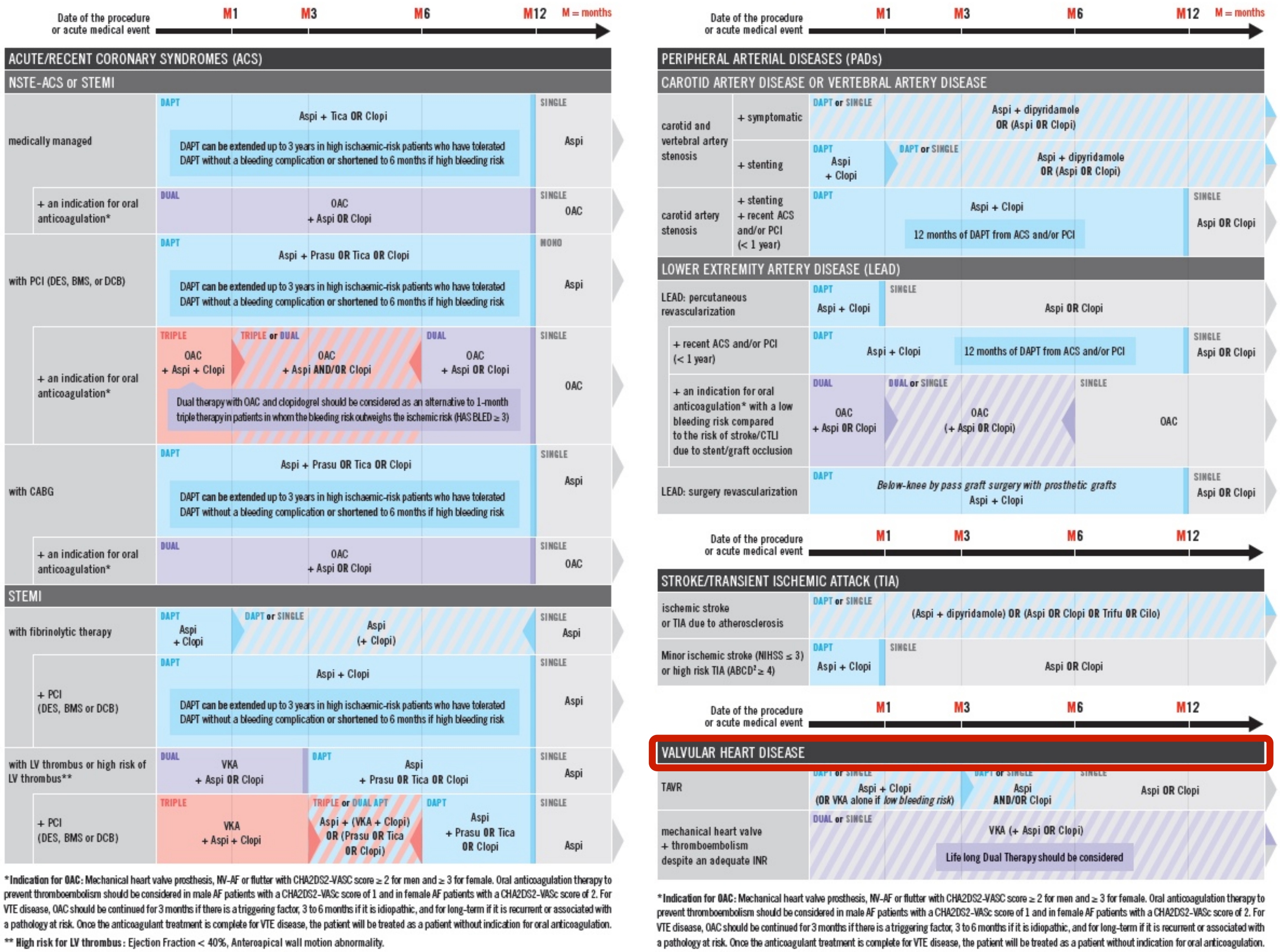

2- Locate the precise clinical situation of your patient (treatment already performed, associated pathologies etc.)

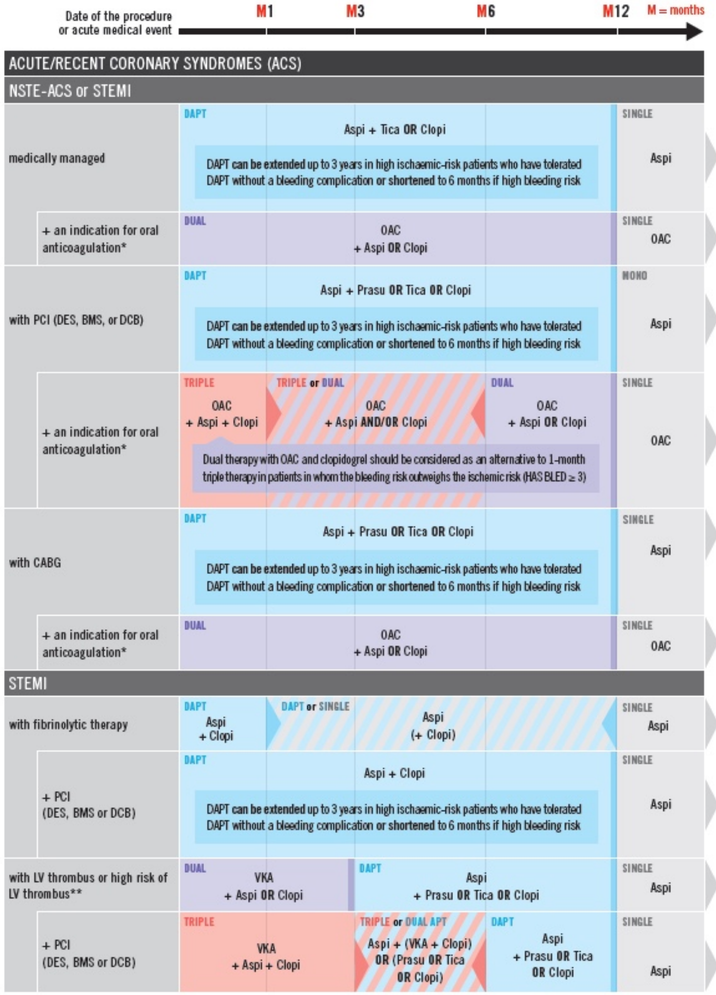

*Indication for OAC: Mechanical heart valve prosthesis, NV-AF or flutter with CHA2DS2-VASC score ≥ 2 for men and ≥ 3 for female. Oral anticoagulation therapy to prevent thromboembolism should be considered in male AF patients with a CHA2DS2-VASc score of 1 and in female AF patients with a CHA2DS2-VASc score of 2. For VTE disease, OAC should be continued for 3 months if there is a triggering factor, 3 to 6 months if it is idiopathic, and for long-term if it is recurrent or associated with a pathology at risk. Once the anticoagulant treatment is complete for VTE disease, the patient will be treated as a patient without indication for oral anticoagulation.

** High risk for LV thrombus: Ejection Fraction < 40%, Anteroapical wall motion abnormality.

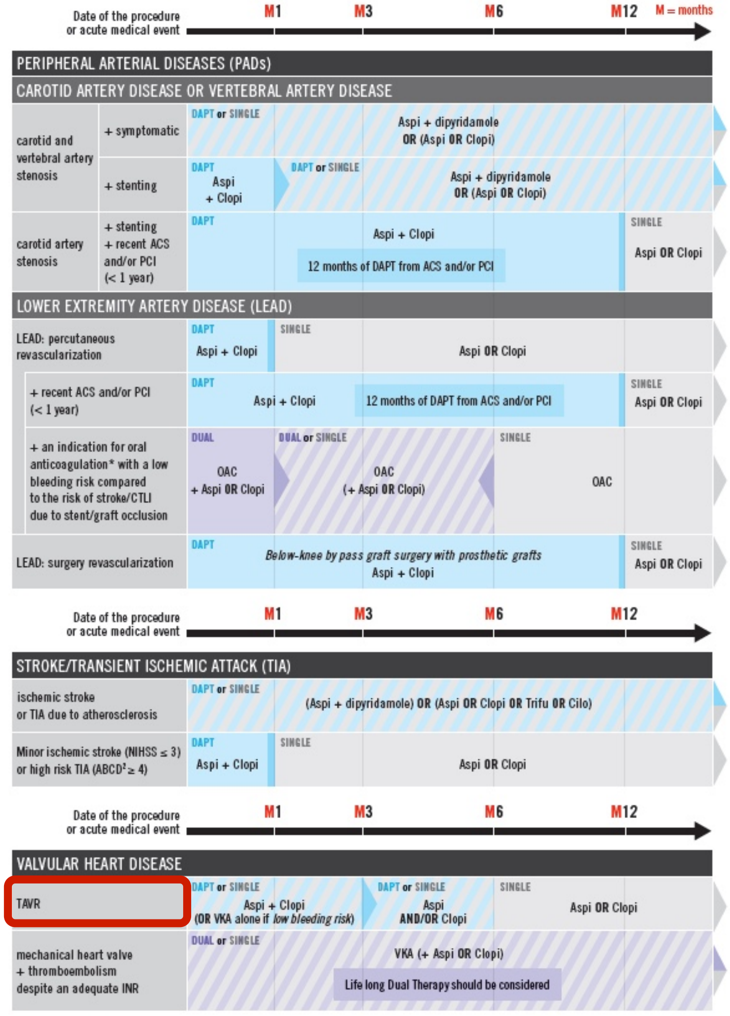

*Indication for OAC: Mechanical heart valve prosthesis, NV-AF or flutter with CHA2DS2-VASC score ≥ 2 for men and ≥ 3 for female. Oral anticoagulation therapy to prevent thromboembolism should be considered in male AF patients with a CHA2DS2-VASc score of 1 and in female AF patients with a CHA2DS2-VASc score of 2. For VTE disease, OAC should be continued for 3 months if there is a triggering factor, 3 to 6 months if it is idiopathic, and for long-term if it is recurrent or associated with a pathology at risk. Once the anticoagulant treatment is complete for VTE disease, the patient will be treated as a patient without indication for oral anticoagulation.

3- In the recommended treatment, find out where your patient is currently (here: 8 months)

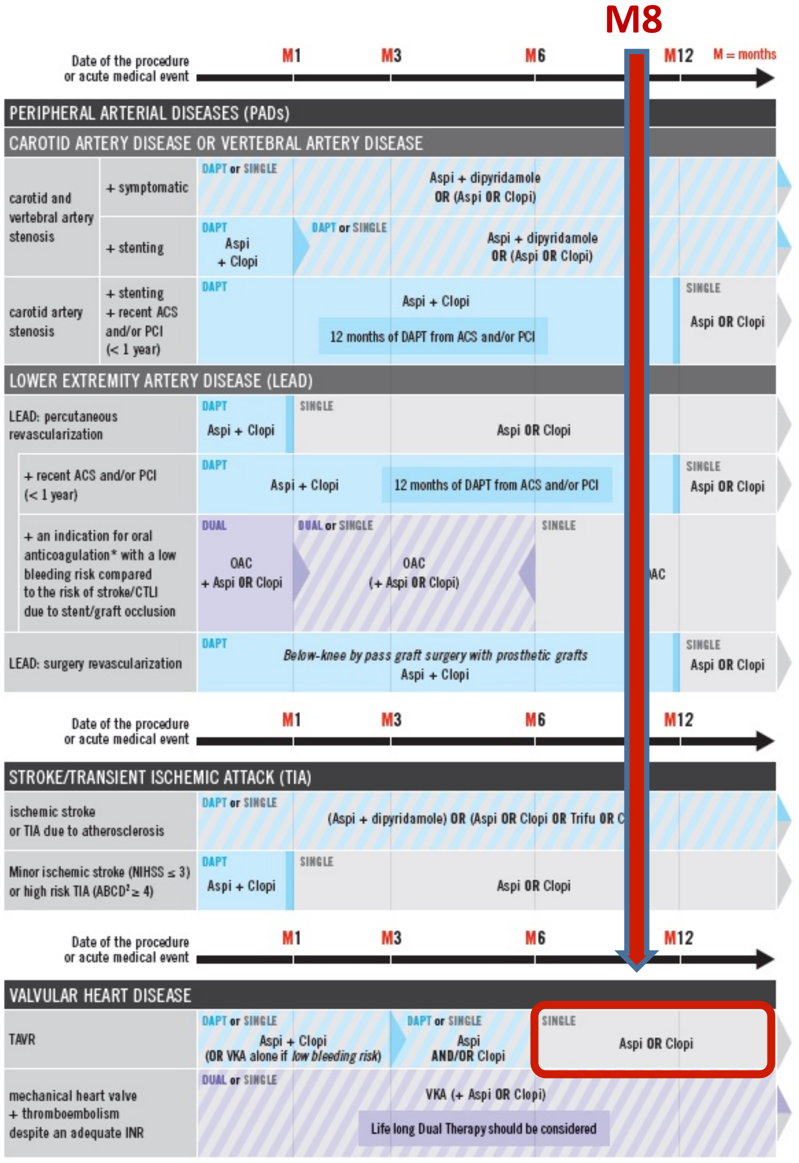

*Indication for OAC: Mechanical heart valve prosthesis, NV-AF or flutter with CHA2DS2-VASC score ≥ 2 for men and ≥ 3 for female. Oral anticoagulation therapy to prevent thromboembolism should be considered in male AF patients with a CHA2DS2-VASc score of 1 and in female AF patients with a CHA2DS2-VASc score of 2. For VTE disease, OAC should be continued for 3 months if there is a triggering factor, 3 to 6 months if it is idiopathic, and for long-term if it is recurrent or associated with a pathology at risk. Once the anticoagulant treatment is complete for VTE disease, the patient will be treated as a patient without indication for oral anticoagulation.

Long-term single antithrombotic therapy is recommended:

1) Aspirin
2) Clopidogrel

4- Check the recommended dosage for the drugs you want to prescribe

SINGLE	Single Therapy: Antiplatelet (SAPT) or Anticoagulation (SACT)
DUAL APT	Dual Antiplatelet Therapy (DAPT)
DUAL	Dual Therapy (SAPT + SACT)
TRIPLE	Triple Therapy (DAPT + SACT)

THERAPY 1 or THERAPY 2	Modulation between two therapies depending on patient condition (according to striped colors)
	Minimum therapy duration
	Maximum therapy duration
	Recommended therapy duration

CLINICAL SITUATIONS NOT FOUND IN THIS TOOL NEED A SPECIALIST'S OPINION

Abbreviations

ABCD² score for TIA Age≥60 years (+1)
Clinical features of the TIA (unilateral weakness (+2), speech disturbance without weakness (+1), other symptoms (0))
Duration of symptoms (< 10 min (0), 10-59 min (+1), ≥60 min (+2))
BP ≥ 140/90 mmHg (+1) Diabetes (+1)

ACS Acute Coronary Syndrome
Aspi Aspirin
BMS Bare-Metal Stent
CABG Coronary Artery By Pass Graft
CHA2DS2-VASc C Congestive Heart failure (+1)
H Hypertension (+1) A2 Age ≥ 75 (+2)
D Diabetes Mellitus (+1) S2 Prior stroke or TIA thromboembolism (+2)
V Vascular disease (+1) A Age 65-74 (+1)
Sc Sex category (i.e.: female sex) (+1)
Cilo Cilostazol
Clopi Clopidogrel
CTLI Chronic Limb-Threatening Ischemia
DAPT Dual Antiplatelet Therapy
DCB Drug-Coated Balloon
DES Drug-Eluting Stent
DOA Direct Oral Anticoagulant
DUAL Dual Therapy: SAPT + SACT
HAS BLED Abnormal renal / liver function (+1 or +2)
Hypertension (+1) Stroke history (+1)
Prior major Bleeding or predisposition to bleeding (+1)
Labile INR (+1) Elderly > 65 (+1)
Drugs (concomitant aspirin, clopidogrel, NSAIDs) or alcohol (+1 or +2)
INR International Normalized Ratio
LEAD Lower Extremity Artery Disease
LV Left Ventricular
NIHSS National Institutes of Health Stroke Scale
NSTE-ACS Non-ST Elevation Acute Coronary Syndrome
NV-AF Non-valvular atrial fibrillation
OAC Oral Anticoagulant : VKA or DOA
PCI Percutaneous coronary intervention (= DES, BMS or DCB)
Prasu Prasugrel
SAPT Single Antiplatelet Therapy
SACT Single Anticoagulation Therapy
SCAD Stable coronary artery disease
STEMI ST-Elevation Myocardial Infarction
TAVR Transcatheter Aortic Valve Replacement
TIA Transient Ischemic Attack
Tica Ticagrelor
Triflu Triflusal
TRIPLE Triple Therapy: DAPT + SACT
VKA Vitamin K Antagonist Transcatheter Aortic Valve
VTE Venous Thromboembolism

TARGET INR FOR MECHANICAL PROSTHESE	Patient-related risk factor[a]	
Prosthesis thrombogenicity	None	≥1
Low[b]	2.5	3.0
Medium[c]	3.0	3.5
High[d]	3.5	4.0

INR = international normalized ratio; LVEF = left ventricular ejection fraction
[a] Mitral or tricuspid valve replacement, previous thromboembolism; atrial fibrillation, mitral stenosis of any degree, LVEF < 35%
[b] Carbomedics, Medtronic Hall, ATS, Medtronic Open-Pivot, St-Jude Medical, On-X, Sorin Bicarbon
[c] Other bileaflet valve with insufficient data
[d] Lillehei-Kaster, Omniscience, Starr-Edwards (ball-cage), Bjork Shiley and other tilting-disc valves

Dosage of antithrombotic drugs

Aspirin: 75-100 mg/day
Aspirin/dipyridamole: 25/200 mg twice a day
Cilostazol: 100 mg twice a day
Clopidogrel: 75 mg/day
Prasugrel: 10 mg/day (5 mg/day in patients with body weight < 60 kg)
Contraindications for prasugrel : previous intracranial haemorrhage, previous ischaemic stroke or transient ischaemic attack, or ongoing bleeds ; prasugrel is not recommended for patients >75 years of age or with a body weight <60 kg.
Ticagrelor: 90 mg twice a day
Contraindications for ticagrelor : previous intracranial haemorrhage or ongoing bleeds.
Triflusal: 600 mg/day
VKA: target INR 2-3 for NV-AF, VTE; LV thrombus
Rivaroxaban (Xarelto):
- Venous thrombo-embolism (venous thrombosis/pulmonary embolism): D1 to D21: 15 mg x 2/day then from D22 onwards: 20 mg/day in a single take
- For the prevention of embolic stroke in patients with NV-AF: 20 mg/day in a single take
- No adjustment on weight, age, sex
- Renal failure
  - Contraindication with creatinine clearance < 15 ml/min
  - With creatinine clearance between 15-49 ml/min:
    - § NV-AF: 15 mg/day
    - § Venous thrombo-embolism: 15 mg x 2/day during the first three weeks then 20 mg/day in a single take
  - No adjustment beyond a creatinine clearance > 50 ml/min

Apixaban (Eliquis):
- For the prevention of embolic stroke in patients with NV-AF:
  - 5 mg x 2/day
  - NV-AF and at least two of the following: age ≥ 80 yo, weight ≤ 60 kg, serum creatinine ≥ 133 micromol/L: 2,5 mg x 2/day
  - With creatinine clearance between 15–29 ml/min: 2,5 mg x 2/day
  - Contraindication with creatinine clearance < 15 ml/min
- Venous thrombo-embolism (venous thrombosis/pulmonary embolism): D1 to D7: 10 mg x 2/day then from D8 onwards: 5 mg x 2/day

Dabigatran (Pradaxa):
- For the prevention of embolic stroke in patients with NV-AF or VTE treatment, after treatment with a parenteral anticoagulant for at least 5 days : 150 mg x 2/day
- 110 mg x 2/day if :
  - § > 80 yo
  - § Patients also treated with Verapamil
  - § clearance between 30–50 ml/min
- Contraindication with creatinine clearance < 30 ml/min

THE COCKCROFT AND GAULT FORMULA (1973)
$C_{Cr} = \{((140 - Age) \times Weight) / (72 \times S_{Cr})\} \times 0.85$ (if female)
C_{Cr} (creatinine clearance) = mL/minute
Age = years Weight = kg S_{Cr} (serum creatinine) = mg/dL

IF BLEEDING DURING DAPT, follow these recommendations *(figure 10)*: https://www.escardio.org/Guidelines/Clinical-Practice-Guidelines/2017-focused-update-on-dual-antiplatelet-therapy-dapt

SPECIFIC CONDITIONS REQUIRING SYSTEMATICALLY AN IN-HOSPITAL SPECIALIST HAVE BEEN EXCLUDED: cancer, auto-immune disease, hemophilia, human immunodeficiency virus (HIV), pediatrics and pregnancy and In-hospital prescriptions (including bridging therapy, perioperative therapy, and treatment of acute phase of cardiovascular event)

1) Aspirin 75-100 mg/day
2) OR Clopidogrel 75 mg/day

In practice

Example 2: two cardiovascular diseases

- At your medical consultation, you meet Mr V, 55 years old (weight: 81 kg, body mass index: 24 kg/m^2).
- Medical history: arterial hypertension (controlled), diabetes, renal failure (creatinine clearance with Cockcroft formula: 30 ml/min) and permanent non-valvular atrial fibrillation
- He had an acute coronary syndrome 5 months ago with a percutaneous coronary intervention (PCI)
- Which antithrombotic therapy is recommended in this clinical situation?

1- Locate in the chapter headings of the tool, the cardiovascular disease of your patient

Date of the procedure or acute medical event → M1 → M3 → M6 → M12 (M = months)

ACUTE/RECENT CORONARY SYNDROMES (ACS)

NSTE-ACS or STEMI

Situation	Date of procedure/event to M12	After M12
medically managed	DAPT: Aspi + Tica OR Clopi<br>DAPT can be extended up to 3 years in high ischaemic-risk patients who have tolerated DAPT without a bleeding complication or shortened to 6 months if high bleeding risk	SINGLE: Aspi
+ an indication for oral anticoagulation*	DUAL: OAC + Aspi OR Clopi	SINGLE: OAC
with PCI (DES, BMS, or DCB)	DAPT: Aspi + Prasu OR Tica OR Clopi<br>DAPT can be extended up to 3 years in high ischaemic-risk patients who have tolerated DAPT without a bleeding complication or shortened to 6 months if high bleeding risk	MONO: Aspi
+ an indication for oral anticoagulation*	TRIPLE (to M1): OAC + Aspi + Clopi<br>TRIPLE or DUAL (M1 to M6): OAC + Aspi AND/OR Clopi<br>DUAL (M6 to M12): OAC + Aspi OR Clopi<br>Dual therapy with OAC and clopidogrel should be considered as an alternative to 1-month triple therapy in patients in whom the bleeding risk outweighs the ischemic risk (HAS BLED ≥ 3)	SINGLE: OAC
with CABG	DAPT: Aspi + Prasu OR Tica OR Clopi<br>DAPT can be extended up to 3 years in high ischaemic-risk patients who have tolerated DAPT without a bleeding complication or shortened to 6 months if high bleeding risk	SINGLE: Aspi
+ an indication for oral anticoagulation*	DUAL: OAC + Aspi OR Clopi	SINGLE: OAC

STEMI

Situation	Date of procedure/event to M12	After M12
with fibrinolytic therapy	DAPT (to M1): Aspi + Clopi<br>DAPT or SINGLE (M1 to M12): Aspi (+ Clopi)	SINGLE: Aspi
+ PCI (DES, BMS or DCB)	DAPT: Aspi + Clopi<br>DAPT can be extended up to 3 years in high ischaemic-risk patients who have tolerated DAPT without a bleeding complication or shortened to 6 months if high bleeding risk	SINGLE: Aspi
with LV thrombus or high risk of LV thrombus**	DUAL (to M3): VKA + Aspi OR Clopi<br>DAPT (M3 to M12): Aspi + Prasu OR Tica OR Clopi	SINGLE: Aspi
+ PCI (DES, BMS or DCB)	TRIPLE (to M3): VKA + Aspi + Clopi<br>TRIPLE or DUAL APT (M3 to M6): Aspi + (VKA + Clopi) OR (Prasu OR Tica OR Clopi)<br>DAPT (M6 to M12): Aspi + Prasu OR Tica OR Clopi	SINGLE: Aspi

* **Indication for OAC:** Mechanical heart valve prosthesis, NV-AF or flutter with CHA2DS2-VASC score ≥ 2 for men and ≥ 3 for female. Oral anticoagulation therapy to prevent thromboembolism should be considered in male AF patients with a CHA2DS2-VASc score of 1 and in female AF patients with a CHA2DS2-VASc score of 2. For VTE disease, OAC should be continued for 3 months if there is a triggering factor, 3 to 6 months if it is idiopathic, and for long-term if it is recurrent or associated with a pathology at risk. Once the anticoagulant treatment is complete for VTE disease, the patient will be treated as a patient without indication for oral anticoagulation.

** **High risk for LV thrombus:** Ejection Fraction < 40%, Anteroapical wall motion abnormality.

2- Locate the precise clinical situation of your patient (treatment already performed, associated pathologies etc.)

Date of the procedure or acute medical event → M1 — M3 — M6 — M12 (M = months)

Situation		Until M12 (timeline M1, M3, M6, M12)	After M12
**ACUTE/RECENT CORONARY SYNDROMES (ACS)**			
**NSTE-ACS or STEMI**			
medically managed		DAPT: Aspi + Tica OR Clopi<br>DAPT can be extended up to 3 years in high ischaemic-risk patients who have tolerated DAPT without a bleeding complication or shortened to 6 months if high bleeding risk	SINGLE: Aspi
	+ an indication for oral anticoagulation*	DUAL: OAC + Aspi OR Clopi	SINGLE: OAC
with PCI (DES, BMS, or DCB)		DAPT: Aspi + Prasu OR Tica OR Clopi<br>DAPT can be extended up to 3 years in high ischaemic-risk patients who have tolerated DAPT without a bleeding complication or shortened to 6 months if high bleeding risk	MONO: Aspi
	+ an indication for oral anticoagulation*	TRIPLE (to M1): OAC + Aspi + Clopi; TRIPLE or DUAL (M1 to M6): OAC + Aspi AND/OR Clopi; DUAL (M6 to M12): OAC + Aspi OR Clopi<br>Dual therapy with OAC and clopidogrel should be considered as an alternative to 1-month triple therapy in patients in whom the bleeding risk outweighs the ischemic risk (HAS BLED ≥ 3)	SINGLE: OAC
with CABG		DAPT: Aspi + Prasu OR Tica OR Clopi<br>DAPT can be extended up to 3 years in high ischaemic-risk patients who have tolerated DAPT without a bleeding complication or shortened to 6 months if high bleeding risk	SINGLE: Aspi
	+ an indication for oral anticoagulation*	DUAL: OAC + Aspi OR Clopi	SINGLE: OAC
**STEMI**			
with fibrinolytic therapy		DAPT (to M1): Aspi + Clopi; DAPT or SINGLE (M1 to M12): Aspi (+ Clopi)	SINGLE: Aspi
	+ PCI (DES, BMS or DCB)	DAPT: Aspi + Clopi<br>DAPT can be extended up to 3 years in high ischaemic-risk patients who have tolerated DAPT without a bleeding complication or shortened to 6 months if high bleeding risk	SINGLE: Aspi
with LV thrombus or high risk of LV thrombus**		DUAL (to M3): VKA + Aspi OR Clopi; DAPT (M3 to M12): Aspi + Prasu OR Tica OR Clopi	SINGLE: Aspi
	+ PCI (DES, BMS or DCB)	TRIPLE (to M3): VKA + Aspi + Clopi; TRIPLE or DUAL APT (M3 to M6): Aspi + (VKA + Clopi) OR (Prasu OR Tica OR Clopi); DAPT (M6 to M12): Aspi + Prasu OR Tica OR Clopi	SINGLE: Aspi

* **Indication for OAC:** Mechanical heart valve prosthesis, NV-AF or flutter with CHA2DS2-VASC score ≥ 2 for men and ≥ 3 for female. Oral anticoagulation therapy to prevent thromboembolism should be considered in male AF patients with a CHA2DS2-VASc score of 1 and in female AF patients with a CHA2DS2-VASc score of 2. For VTE disease, OAC should be continued for 3 months if there is a triggering factor, 3 to 6 months if it is idiopathic, and for long-term if it is recurrent or associated with a pathology at risk. Once the anticoagulant treatment is complete for VTE disease, the patient will be treated as a patient without indication for oral anticoagulation.

** **High risk for LV thrombus:** Ejection Fraction < 40%, Anteroapical wall motion abnormality.

2bis- Locate the precise clinical situation of your patient (treatment already performed, associated pathologies etc.)

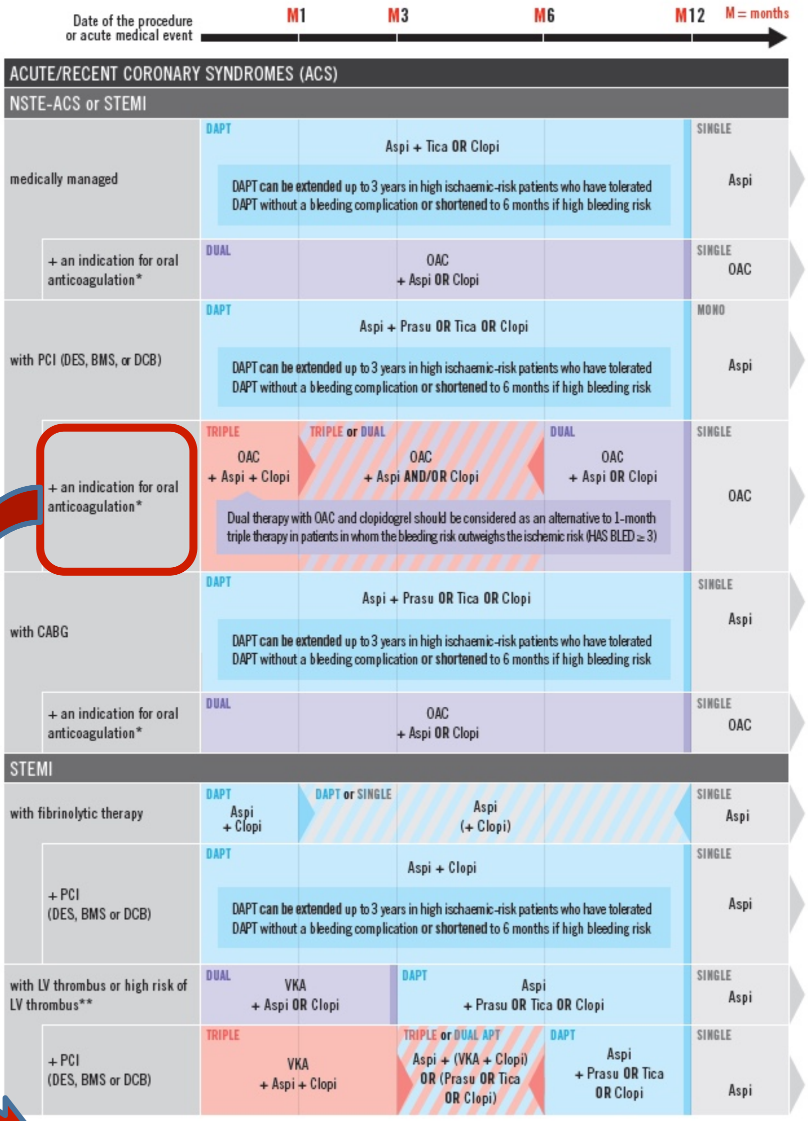

ABBREVIATIONS

CHA2DS2-VASc **C** Congestive Heart failure (+1)
H Hypertension (+1) **A2** Age ≥ 75 (+2)
D Diabetes Mellitus (+1) **S2** Prior stroke or TIA thromboembolism (+2)
V Vascular disease (+1) **A** Age 65-74 (+1)
Sc Sex category (i.e.: female sex) (+1)

Hypertension and diabetes = 2 points
→ Indication for oral anticoagulation

HAS BLED **A**bnormal renal / liver function (+1 or +2)
Hypertension (+1) **S**troke history (+1)
Prior major **B**leeding or predisposition to bleeding (+1)
Labile INR (+1) **E**lderly > 65 (+1)
Drugs (concomitant aspirin, clopidogrel, NSAIDs) or alcohol (+1 or +2)

Abnormal renal function = 1 point
Drugs = 1 point
HAS BLED = 2

* **Indication for OAC :** Mechanical heart valve prosthesis, NV-AF or flutter with CHA2DS2-VASC score ≥ 2 for men and ≥ 3 for female. Oral anticoagulation therapy to prevent thromboembolism should be considered in male AF patients with a CHA2DS2-VASc score of 1 and in female AF patients with a CHA2DS2-VASc score of 2. For

3- In the recommended treatment, find out where your patient is currently (here: 5 months)

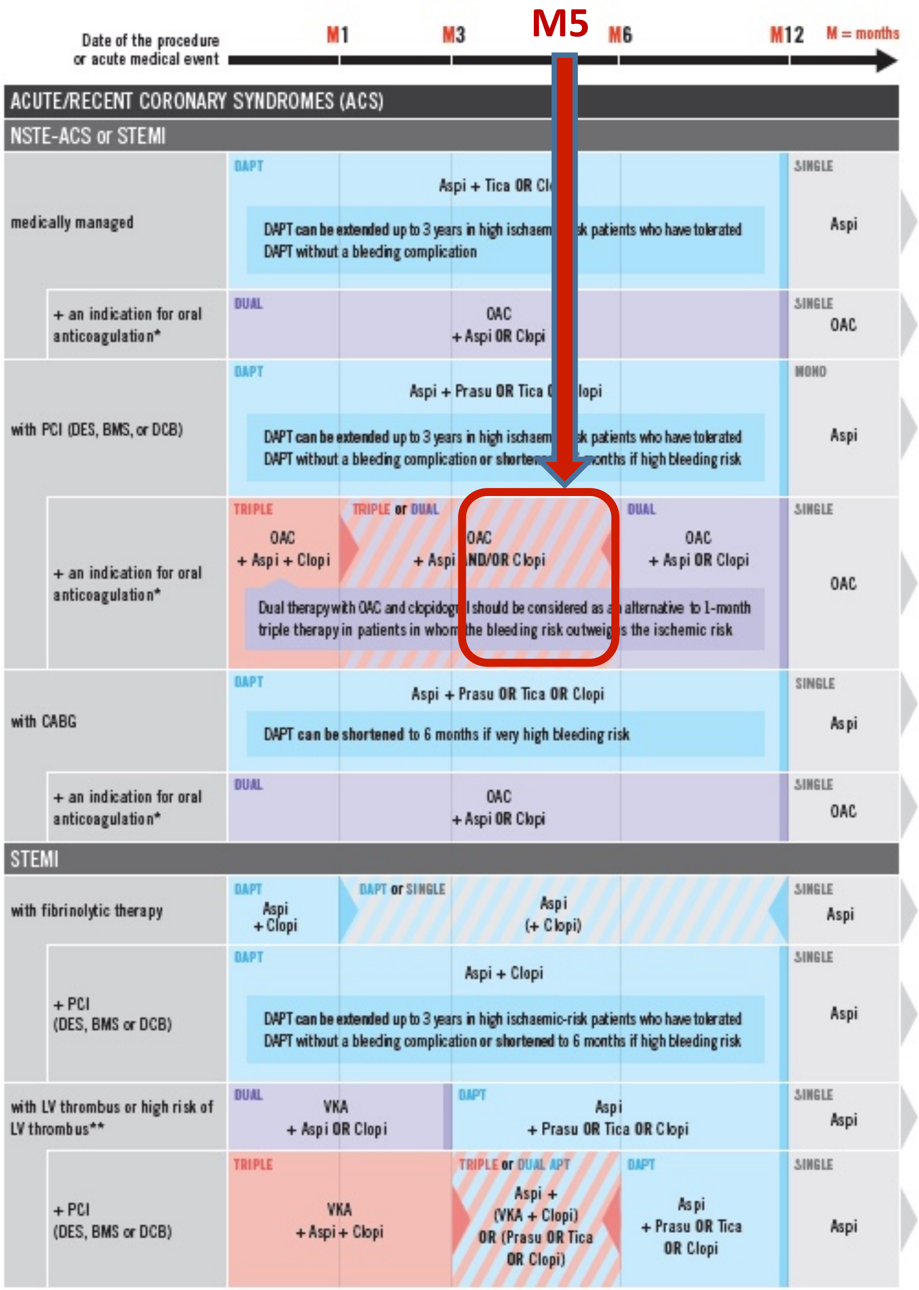

a pathology at risk. Once the anticoagulant treatment is complete for VTE disease, the patient will be treated as a patient without indication for oral anticoagulation.

** **High risk for LV thrombus**: Ejection Fraction < 40%, Anteroapical wall motion abnormality.

Here, two options are possible according to the ischemic and bleeding risk of your patient:

1) Dual therapy: OAC + Aspirin OR Clopidogrel up to 12 months (so for another 7 months)
2) Triple therapy: OAC + Aspirin + Clopidogrel up to 6 months (so for another 1 month) and then a dual therapy with OAC + Aspirin OR Clopidogrel up to 12 months (so for another 6 months)

4- Check the recommended dosage for the drugs you want to prescribe

SINGLE	Single Therapy: Antiplatelet (SAPT) or Anticoagulation (SACT)
DUAL APT	Dual Antiplatelet Therapy (DAPT)
DUAL	Dual Therapy (SAPT + SACT)
TRIPLE	Triple Therapy (DAPT + SACT)

THERAPY 1 or THERAPY 2	Modulation between two therapies depending on patient condition (according to striped colors)
▶	Minimum therapy duration
◀	Maximum therapy duration
|	Recommended therapy duration

CLINICAL SITUATIONS NOT FOUND IN THIS TOOL NEED A SPECIALIST'S OPINION

Abbreviations

ABCD² score for TIA Age≥60 years (+1)
Clinical features of the TIA (unilateral weakness (+2), speech disturbance without weakness (+1), other symptoms (0))
Duration of symptoms (< 10 min (0), 10-59 min (+1), ≥60 min (+2))
BP ≥ 140/90 mmHg (+1) Diabetes (+1)

ACS Acute Coronary Syndrome
Aspi Aspirin
BMS Bare-Metal Stent
CABG Coronary Artery By Pass Graft
CHA2DS2-VASc C Congestive Heart failure (+1)
H Hypertension (+1) A2 Age ≥ 75 (+2)
D Diabetes Mellitus (+1) S2 Prior stroke or TIA thromboembolism (+2)
V Vascular disease (+1) A Age 65-74 (+1)
Sc Sex category (i.e.: female sex) (+1)
Cilo Cilostazol
Clopi Clopidogrel
CTLI Chronic Limb-Threatening Ischemia
DAPT Dual Antiplatelet Therapy
DCB Drug-Coated Balloon
DES Drug-Eluting Stent
DOA Direct Oral Anticoagulant
DUAL Dual Therapy: SAPT + SACT
HAS BLED Abnormal renal / liver function (+1 or +2)
Hypertension (+1) Stroke history (+1)
Prior major Bleeding or predisposition to bleeding (+1)
Labile INR (+1) Elderly > 65 (+1)
Drugs (concomitant aspirin, clopidogrel, NSAIDs) or alcohol (+1 or +2)
INR International Normalized Ratio
LEAD Lower Extremity Artery Disease
LV Left Ventricular
NIHSS National Institutes of Health Stroke Scale
NSTE-ACS Non-ST Elevation Acute Coronary Syndrome
NV-AF Non-valvular atrial fibrillation
OAC Oral Anticoagulant: VKA or DOA
PCI Percutaneous coronary intervention (= DES, BMS or DCB)
Prasu Prasugrel
SAPT Single Antiplatelet Therapy
SACT Single Anticoagulation Therapy
SCAD Stable coronary artery disease
STEMI ST-Elevation Myocardial Infarction
TAVR Transcatheter Aortic Valve Replacement
TIA Transient Ischemic Attack
Tica Ticagrelor
Triflu Triflusal
TRIPLE Triple Therapy: DAPT + SACT
VKA Vitamin K Antagonist Transcatheter Aortic Valve
VTE Venous Thromboembolism

TARGET INR FOR MECHANICAL PROSTHESE	Patient-related risk factor [a]	
Prosthesis thrombogenicity	None	≥1
Low[b]	2.5	3.0
Medium[c]	3.0	3.5
High[d]	3.5	4.0

INR = international normalized ratio; LVEF = left ventricular ejection fraction
[a] Mitral or tricuspid valve replacement, previous thromboembolism; atrial fibrillation, mitral stenosis of any degree, LVEF < 35%
[b] Carbomedics, Medtronic Hall, ATS, Medtronic Open-Pivot, St-Jude Medical, On-X, Sorin Bicarbon
[c] Other bileaflet valve with insufficient data
[d] Lillehei-Kaster, Omniscience, Starr-Edwards (ball-cage), Bjork Shiley and other tilting-disc valves

Dosage of antithrombotic drugs

Aspirin: 75-100 mg/day
Aspirin/dipyridamole: 25/200 mg twice a day
Cilostazol: 100 mg twice a day
Clopidogrel: 75 mg/day
Prasugrel: 10 mg/day (5 mg/day in patients with body weight < 60 kg)
Contraindications for prasugrel : previous intracranial haemorrhage, previous ischaemic stroke or transient ischaemic attack, or ongoing bleeds ; prasugrel is not recommended for patients >75 years of age or with a body weight <60 kg.
Ticagrelor: 90 mg twice a day
Contraindications for ticagrelor : previous intracranial haemorrhage or ongoing bleeds.
Triflusal: 600 mg/day
VKA: target INR 2-3 for NV-AF, VTE; LV thrombus
Rivaroxaban (Xarelto):
- Venous thrombo-embolism (venous thrombosis/pulmonary embolism): D1 to D21: 15 mg x 2/day then from D22 onwards: 20 mg/day in a single take
- For the prevention of embolic stroke in patients with NV-AF: 20 mg/day in a single take
- No adjustment on weight, age, sex
- Renal failure
  - Contraindication with creatinine clearance < 15 ml/min
  - With creatinine clearance between 15-49 ml/min:
    - § NV-AF: 15 mg/day
    - § Venous thrombo-embolism: 15 mg x 2/day during the first three weeks then 20 mg/day in a single take
  - No adjustment beyond a creatinine clearance > 50 ml/min

Apixaban (Eliquis):
- For the prevention of embolic stroke in patients with NV-AF:
  - 5 mg x 2/day
  - NV-AF and at least two of the following: age ≥ 80 yo, weight ≤ 60 kg, serum creatinine ≥ 133 micromol/L: 2,5 mg x 2/day
  - With creatinine clearance between 15–29 ml/min: 2,5 mg x 2/day
  - Contraindication with creatinine clearance < 15 ml/min
- Venous thrombo-embolism (venous thrombosis/pulmonary embolism): D1 to D7: 10 mg x 2/day then from D8 onwards: 5 mg x 2/day

Dabigatran (Pradaxa):
- For the prevention of embolic stroke in patients with NV-AF or VTE treatment, after treatment with a parenteral anticoagulant for at least 5 days : 150 mg x 2/day
- 110 mg x 2/day if :
  - § > 80 yo
  - § Patients also treated with Verapamil
  - § clearance between 30–50 ml/min
- Contraindication with creatinine clearance < 30 ml/min

THE COCKCROFT AND GAULT FORMULA (1973)
$C_{Cr} = \{((140 - Age) \times Weight) / (72 \times S_{Cr})\} \times 0.85$ (if female)
C_{Cr} (creatinine clearance) = mL/minute
Age = years Weight = kg S_{Cr} (serum creatinine) = mg/dL

IF BLEEDING DURING DAPT, follow these recommendations *(figure 10)*:
https://www.escardio.org/Guidelines/Clinical-Practice-Guide-lines/2017-focused-update-on-dual-antiplatelet-therapy-dapt

SPECIFIC CONDITIONS REQUIRING SYSTEMATICALLY AN IN-HOSPITAL SPECIALIST HAVE BEEN EXCLUDED: cancer, auto-immune disease, hemophilia, human immunodeficiency virus (HIV), pediatrics and pregnancy and In-hospital prescriptions (including bridging therapy, perioperative therapy, and treatment of acute phase of cardiovascular event)

OAC :
- VKA with a target INR: 2–3
- Rivaroxaban 15 mg/day
- Apixaban 5 mg X 2/day
- Dabigatran is contraindicated

Antiplatelets:
- Aspirin 75-100 mg/day
- Clopidogrel 75 mg/day
