# Supplementary material for: A prescription support-tool for chronic management of oral antithrombotic combinations in adults based on a systematic review of international guidelines
Source: PLoS One. 2019 Feb 14;14(2):e0211695. doi: 10.1371/journal.pone.0211695 (PMC6375571; doi:10.1371/journal.pone.0211695)
Supplement: S1 Table — (DOCX) [file pone.0211695.s004.docx]

**S1 Table: Synthesis of recommendations from all selected guidelines dealing with the use of oral antithrombotic (AT) drugs: indications, duration and dosage in adults**

| **Indications** | **Single antiplatelet therapy**  **(SAPT)** | **Dual antiplatelet therapy**  **(DAPT)** | **Single anticoagulation therapy (SACT)** | **Dual therapy:**  **SAPT + SACT** | **Triple therapy:**  **DAPT + SACT** |
| --- | --- | --- | --- | --- | --- |
| **CORONARY ARTERY DISEASE (CAD)** | | | | | |
| **Stable ischemic heart disease (SIHD)** | | | | | |
| **SIHD with PCI: DES/BMS or DCB** | **2. Life-long SAPT**  [1-16]  **Aspirin** 75–100 mg/day  **USA: Ia**  **Europe: Ia** | **1. DAPT for at least 6 months (6–12 months) if at low risk of bleeding**  [1-3,9,11,14-16]  **No high bleeding risk**  **First 6 months**   - **USA: I** - **Europe: Ia**   **After 6 months**   - **USA: IIb** - **Europe: IIb**   **Aspirin** 75–100 mg/day  **AND**  **Clopidogrel** 75 mg/day  DAPT can be extended to 30 months if no bleeding risk or shortened to 1–3 months if high risk of bleeding  [1,3,9,14,16]  **High bleeding risk**   - **USA: IIb** - **Europe IIa** |  |  |  |
| **SIHD with CABG** | **2. Life-long SAPT**  [1-15]  **Aspirin** 75–100 mg/day  **USA: Ia**  **Europe: Ia** | **1. DAPT for 12 months (only for Americans)**  [1,16]  **USA: IIb**  **Europe: no grade (no indication for DAPT unless concomitant or prior indication overrides)**  **Aspirin** 75–100 mg/day  **AND**  **Clopidogrel** 75 mg/day |  |  |  |
| **SIHD**  **AND**  **NV-AF**  **CHA2DS2-VASc score ≥ 2 for males and ≥ 3 for females** |  |  | **Life-long SACT**  [9,14,16,18-24]  **USA: weak recommendation**  **Europe: Ia** |  |  |
| **SIHD with PCI**  **AND**  **NV-AF CHA2DS2-VASc score ≥ 2 for males and ≥ 3 for females** |  |  | **3. Life-long SACT**  [9,14,16,18-24]  **USA: Ib**  **Europe: Ib** | **2. Dual therapy up to 12 months**  [9,14,16,18-24]  **OAC**  **AND**  **Aspirin** 75–100 mg/day  **OR**  **Clopidogrel** 75–100 mg/day  **Dual therapy** with clopidogrel and OAC should be considered as an alternative to 1-month triple therapy in patients in whom the bleeding risk outweighs the ischemic risk  [14,16,19,20,21,23,24]  **USA: “weak recommendation”**  **Europe: IIa** | **1. Triple therapy for 1–6 months**  [9,16,18-21,23,24]  **OAC**  **AND**  **Aspirin** 75–100 mg/day  **AND**  **Clopidogrel** 75–100 mg/day  **USA: “weak recommendation”**  **Europe: IIa** |
| **SIHD with PCI**  **AND**  **Mechanical heart valve** |  |  | **3. Life-long SACT**  [9,14,16,19,25]  **USA: Ib**  **Europe: Ib** | **2. Dual therapy**  **up to 12 months**  [7,9,14,16,19,25]  **VKA**  **AND**  **Clopidogrel** 75mg/day  **Dual therapy** should be considered an alternative to 1-month triple therapy in patients in whom the bleeding risk outweighs the ischemic risk  [14,16,19,25]  **Life-long dual therapy may be considered** for patients with low risk of bleeding: “the addition of low dose aspirin to VKA may be considered in the case of concomitant atherosclerotic disease” [25,26]  **USA: “weak recommendation”**  **Europe: IIa** | **1. Triple therapy for 1-6 months**  [7,14,16,19,25]  **VKA**  **AND**  **Aspirin** 75–100 mg/day  **AND**  **Clopidogrel** 75–100 mg/day  **USA: “weak recommendation”**  **Europe: IIa** |
| **ACUTE/RECENT CORONARY SYNDROMES (ACS):**  **NSTE-ACS (Non-ST Elevation Acute Coronary Syndrome)** | | | | | |
| **NSTE-ACS medically managed** | **2. Life-long SAPT**  [1,4,5,7,8,10-13,15,16,27-29]  **Aspirin** 75–100 mg/day  **USA: Ia**  **Europe: Ia** | **1. DAPT for at least 12 months**  [1,4,8,10-13,15,16,27-29]  **Aspirin** 75–100 mg/day  **AND**  **Ticagrelor** 90 mg twice a day  **OR**  **Clopidogrel** 75 mg/day if these are not available or are contraindicated  DAPT can be extended to 3 years in high ischemic-risk patients who have tolerated DAPT without a bleeding complication or shortened to 6 months if high risk of bleeding  [1,16,27]  **No high bleeding risk**  **First 6 months**   - **USA: Ib** - **Europe: Ia**   **After 6 months**   - **USA: IIb** - **Europe: IIb**   **High bleeding risk**   - **USA: IIb** - **Europe IIa** |  |  |  |
| **NSTE-ACS with PCI: DES/BMS** | **2. Life-long SAPT**  [1,4,5,7,8,10-16,27-29]  **Aspirin** 75–100 mg/day  **USA: Ia**  **Europe: Ia** | **1. DAPT should be given for at least 12 months**  [1,4,8,10-16,27-29]  **Aspirin** 75–100 mg/day  **AND**  **A potent P2Y12 inhibitor**  **Prasugrel** 10 mg/day (5 mg/day in patients with body weight < 58 kg)  **OR**  **Ticagrelor** 90 mg twice a day  **OR**  **Clopidogrel** 75 mg/day if these are not available or are contraindicated  DAPT can be extended to 3 years in high ischemic-risk patients who have tolerated DAPT without bleeding complications or shortened to 6 months if high risk of bleeding  [1,14,16,27]  **No high bleeding risk**  **First 12 months**   - **USA: I** - **Europe: Ia**   **After 12 months**   - **USA: IIb** - **Europe: IIb**   **High bleeding risk (6 months)**   - **USA: IIb** - **Europe IIa** |  |  |  |
| **NSTE-ACS with CABG** | **2. Life-long SAPT**  [1,4,5,7,8,10-16,27-29]  **Aspirin** 75–100 mg/day  **USA: Ia**  **Europe: Ia** | **1. DAPT should be given for 12 months**  [1,4,8,10,11,13,16,27-29]  **Aspirin** 75–100 mg/day  **AND**  **A potent P2Y12 inhibitor**  **Prasugrel** 10 mg/day (5 mg/day in patients with body weight < 58 kg)  **OR**  **Ticagrelor** 90 mg twice a day  **OR**  **Clopidogrel** 75 mg/day if these are not available or are contraindicated  DAPT can be shortened to 6 months if high risk of bleeding [1,16,27]  **No high bleeding risk**  **First 12 months**   - **USA: Ic** - **Europe: Ic**   **After 12 months**   - **USA:** - **Europe: IIb**   **High bleeding risk (6 months)**   - **USA: IIb** - **Europe IIa** |  |  |  |
| **NSTE-ACS medically managed OR with CABG**  **AND**  **NV-AF CHA2DS2-VASc score ≥ 2 for males and ≥ 3 for females** |  |  | **2. Life-long SACT**  [5,18,20,27]  **USA: no grade**  **Europe: IIa** | **1. Dual therapy for 12 months**  [5,18,20,27]  **OAC**  **AND**  **Aspirin** 75–100 mg/day  **OR**  **Clopidogrel** 75–100 mg/day  **USA: no grade**  **Europe: IIa** |  |
| **NSTE-ACS with PCI**  **AND**  **NV-AF CHA2DS2-VASc score ≥ 2 for males and ≥ 3 for females** |  |  | **3. Life-long SACT**  [4,5,14,16,18-21,23,24,27]  **USA: “weak recommendation”**  **Europe: IIa** | **2. Dual therapy up to 12 months**  [4,5,14,16,18-21,23,24,27]  **OAC**  **AND**  **Aspirin** 75–100 mg/day  **OR**  **Clopidogrel** 75–100 mg/day  Dual therapy with OAC and clopidogrel 75 mg/day may be considered an alternative to triple antithrombotic therapy in selected patients (HAS-BLED ≥3 and low risk of stent thrombosis). [16,19-21,23,24,27]  **USA: “weak recommendation”**  **Europe: IIa** | **1. Triple therapy for 1-6 months**  [14,16,18-21,23,24,27,28]  **OAC**  **AND**  **Aspirin** 75–100 mg/day  **AND**  **Clopidogrel** 75–100 mg/day  In case of very high bleeding risk, triple therapy can be reduced to 1 month[16,19,24,27]  **USA: “weak recommendation”**  **Europe: IIa** |
| **NSTE-ACS with PCI**  **AND**  **Mechanical Heart Valve** |  |  | **3. Life-long SACT**  [4,14,16,25]  **USA:**  **Europe: IIa** | **2. Dual therapy**  **up to 12 months**  [4,5,7,14,16,25]  **VKA**  **AND**  **Clopidogrel** 75mg/day  **Dual therapy** should be considered an alternative to 1-month triple therapy in patients in whom the bleeding risk outweighs the ischaemia risk  [16,19,25]  **Life-long dual therapy may be considered** for patients with a low risk of bleeding “the addition of low dose aspirin to VKA may be considered in the case of concomitant atherosclerotic disease”  [17,25,26]  **USA:**  **Europe: IIa** | **1. Triple therapy for 1-6 months**  [7,14,16,25,28]  **VKA**  **AND**  **Aspirin** 75–100 mg/day  **AND**  **Clopidogrel** 75–100 mg/day  **USA:**  **Europe: IIa** |
| **ACUTE/RECENT CORONARY SYNDROMES (ACS):**  **STEMI (ST-elevation Myocardial Infarction)** | | | | | |
| **STEMI medically managed** | **2. Life-long SAPT**  [1,4,5,7,8,10-13,15,16,30-32]  **Aspirin** 75–100 mg/day  **USA: Ia**  **Europe: Ia** | **1. DAPT should be given for at least 12 months**  [1,4,5,8,10,11, 12,13,15,16,30,32]  **Aspirin** 75–100 mg/day  **AND**  **Clopidogrel** 75 mg/day  **OR**  **Ticagrelor** 90 mg twice a day  **USA: I**  **Europe: IIa**  DAPT can be extended to 3 years in high ischemic-risk patients who have tolerated DAPT without a bleeding complication or shortened to 6 months if high risk of bleeding  [1,16,30]  **USA: IIb**  **Europe: IIb** |  |  | In low bleeding-risk patients who receive aspirin and clopidogrel, low-dose rivaroxaban (2.5 mg twice daily) may be considered[30]  **Europe: IIb** |
| **STEMI with PCI: DES/BMS** | **2. Life-long SAPT**  [1,4,5,7,8,10-16,30-32]  **Aspirin** 75–100 mg/day  **USA: Ia**  **Europe: Ia** | **1. DAPT should be given for at least 12 months**  [1,4,8,10-16,30-32]  **Aspirin** 75–100 mg/day  **AND**  **A potent P2Y12 inhibitor**  **Prasugrel** 10 mg/day (5 mg/day in patients with body weight < 58 kg)  **OR**  **Ticagrelor** 90 mg twice a day  **OR**  **Clopidogrel** 75 mg/day if these are not available or are contraindicated  DAPT can be extended to 3 years in high ischemic-risk patients who have tolerated DAPT without bleeding complications or shortened to 6 months if high risk of bleeding [1,14,16,30]  **No high bleeding risk**  **First 12 months**   - **USA: I** - **Europe: Ia**   **After 12 months**   - **USA: IIb** - **Europe: IIb**   **High bleeding risk (6 months)**  **USA: IIb**  **Europe IIa** |  |  | In low bleeding-risk patients who receive aspirin and clopidogrel, low-dose rivaroxaban (2.5 mg twice daily) may be considered[30]  **Europe: IIb** |
| **STEMI with CABG** | **2. Life-long SAPT**  [1,4,5,7,8,10-16,30-32]  **Aspirin** 75–100 mg/day  **USA: Ia**  **Europe: Ia** | **1. DAPT should be given for at least 12 months** [1,4,5,8,10,11,13,16,32]  **Aspirin** 75–100 mg/day  **AND**  **A potent P2Y12 inhibitor**  **Prasugrel:** 10 mg/day (5 mg/day in patients with body weight < 58 kg)  **OR**  **Ticagrelor:** 90 mg twice a day  **OR**  **Clopidogrel** 75 mg/day if these are not available or are contraindicated  **USA: I**  **Europe: I**  DAPT can be extended to 3 years in high ischemic-risk patients who have tolerated DAPT without bleeding complications or shortened to 6 months if high risk of bleeding  [1,16,30]  **USA: IIb**  **Europe: IIb** |  |  | In low bleeding-risk patients who receive aspirin and clopidogrel, low-dose rivaroxaban (2.5 mg twice daily) may be considered[30]  **Europe: IIb** |
| **STEMI medically managed**  **AND**  **NV-AF CHA2DS2-VASc score ≥ 2 for males and ≥ 3 for females** |  |  | **2. Life-long SACT** [5,18,20]  **USA:**  **Europe: IIa** | **1. Dual therapy for 12 months**  [5,18,20]  **OAC**  **AND**  **Clopidogrel** 75–100 mg/day  **USA:**  **Europe: IIa** |  |
| **STEMI with PCI**  **AND**  **NV-AF CHA2DS2-VASc score ≥ 2 for males and ≥ 3 for females** |  |  | **3. Life-long SACT** [4,5,14,16,18-21,23-25]  **USA: weak recommendation**  **Europe: IIa** | **2. Dual therapy up to 12 months**  [5,14,16,18-21,23,24,30,33,34]  **OAC**  **AND**  **Aspirin** 75–100 mg/day  **OR**  **Clopidogrel** 75–100 mg/day  **Dual therapy** with clopidogrel and OAC should be considered as an alternative to 1-month triple therapy in patients in whom the bleeding risk outweighs the ischemic risk [16,19-21,23,24]  **USA: weak recommendation**  **Europe: IIa** | **1. Triple therapy for 1-6 months**  [7,14,16,18-21,23,24,30]  **OAC**  **AND**  **Aspirin** 75–100 mg/day  **AND**  **Clopidogrel** 75–100 mg/day  In case of very high bleeding, triple therapy **can be reduced to 1 month**  [16,19,23,24]  **USA: weak recommendation**  **Europe: IIa** |
| **STEMI with PCI**  **AND**  **Mechanical heart valve** |  |  | **3. Life-long SACT**  [4,5,14,16,19,25]  **USA:**  **Europe: IIa** | **2. Dual therapy up to 12 months**  [4,5,7,14,16,19,25]  **VKA**  **AND**  **Clopidogrel** 75mg/day  **Dual therapy** should be considered as an alternative to 1-month triple therapy in patients in whom the bleeding risk outweighs the ischemic risk  [16,19,25]  **Life-long dual therapy may be considered** for patients with a low risk of bleeding “the addition of low dose aspirin to VKA may be considered in the case of concomitant atherosclerotic disease” [25,26]  **USA:**  **Europe: IIa** | **1. Triple therapy for 1-6 months**  [14,16,19,25,30]  **VKA**  **AND**  **Aspirin** 75–100 mg/day  **AND**  **Clopidogrel** 75–100 mg/day  **USA:**  **Europe: IIa** |
| **STEMI with fibrinolytic therapy but without PCI** | **2. Life-long SAPT** [1,4,5,7,8,10-13,15,30-32]  **Aspirin** 75–100 mg/day  **USA: Ia**  **Europe: Ia** | **1. DAPT should be given for at least 1 month and ideally 12 months**  [1,5,8,10-13,30-32]  **Aspirin 75–100 mg/day**  **AND**  **Clopidogrel** 75 mg/day  **USA: I**  **Europe: Ic**  “Expanding the duration of DAPT up to 12 months should be considered in these patients”  “Ideally at least 12 months” [1,30]  **USA: IIb**  **Europe: IIb** |  |  | In low bleeding-risk patients who receive aspirin and clopidogrel, low-dose rivaroxaban (2.5 mg twice daily) may be considered[30]  **Europe: IIb** |
| **STEMI with fibrinolytic therapy before PCI** | **2. Life-long SAPT**  [1,4,5,7,8,10-13,15,30-32]  **Aspirin** 75–100 mg/day  **USA: Ia**  **Europe: Ia** | **1. DAPT should be given for at least 12 months**  [1,8, 10-13,30-32]  **Aspirin** 75–100 mg/day  **AND**  **Clopidogrel** 75 mg/day  **USA: I**  **Europe: Ic**  DAPT can be extended to 3 years in high ischemic-risk patients who have tolerated DAPT without bleeding complications or shortened to 6 months if high risk of bleeding  [1,30]  **USA: IIb**  **Europe: IIb** |  |  | In low bleeding-risk patients who receive aspirin and clopidogrel, low-dose rivaroxaban (2.5 mg twice daily) may be considered[30]  **Europe: IIb** |
| **STEMI medically managed without PCI**  **AND**  **LV thrombus or at high risk of LV thrombus** | **3. Life-long SAPT** [4,5,7,8,12,30-32]  **Aspirin** 75–100 mg/day  **USA:**  **Europe: Ia** | **2. DAPT up to 12 months**  [8,12]  **Aspirin** 75–100 mg/day  **AND**  **A potent P2Y12 inhibitor**  **Prasugrel:** 10 mg/day (5 mg/day in patients with body weight < 58 kg)  **OR**  **Ticagrelor:** 90 mg twice a day  **OR**  **Clopidogrel** 75 mg/day if these are not available or are contraindicated  **USA:**  **Europe: IIa** |  | **1. Dual therapy for 3 months**  [7,12,30,31,33]    **VKA**  **AND**  **Aspirin** 75–100 mg/day  **USA:**  **Europe: IIa** |  |
| **STEMI with PCI**  **AND**  **LV thrombus or at high risk of LV thrombus** | **3. Life-long SAPT**  [4,5,7,8,12,14,30-32]  **Aspirin** 75–100 mg/day  **USA:**  **Europe: Ia** | **2. DAPT up to 12 months** [8,12,14]  **Aspirin** 75–100 mg/day  **AND**  **A potent P2Y12 inhibitor**  **Prasugrel:** 10 mg/day (5 mg/day in patients with body weight < 58 kg)  **OR**  **Ticagrelor:** 90 mg twice a day  **OR**  **Clopidogrel** 75 mg/day if these are not available or are contraindicated  **USA:**  **Europe: IIa** |  |  | **1. Triple therapy for**  **3–6 months**[7,12,14,30,31,33]  **OAC**  **AND**  **Aspirin** 75–100 mg/day  **AND**  **Clopidogrel** 75–100 mg/day  **USA:**  **Europe: IIa** |

| **NON VALVULAR ATRIAL FIBRILLATION (NV-AF) AND ATRIAL FLUTTER**[19,34]  **(Ongoing management with antithrombotic therapy is recommended with atrial flutter to align with recommended antithrombotic therapy for patients with AF)** | | | | | | |
| --- | --- | --- | --- | --- | --- | --- |
| **NV-AF**  **Males: CHA2DS2-VASc score = 1**  **Females: CHA2DS2-VASc score = 2** |  |  | **Life-long SACT**  **should be considered**  [11,17-24,33,35-43]  **USA: IIa**  **Europe: IIa** |  |  | |
| **NV-AF**  **Males: CHA2DS2-VASc score ≥ 2**  **Females:**  **CHA2DS2-VASc score ≥ 3** |  |  | **Life-long SACT**  [11,17-24,33,35-37,39-44]  **USA: Ia**  **Europe: Ia** |  |  | |
| **NV-AF CHA2DS2-VASc score ≥ 1 for males and ≥ 2 for females**  **AND surgical occlusion or exclusion of the LAA** |  |  | **Life-long SACT** in at-risk patients with AF for stroke prevention  [20,44]  **USA:**  **Europe: Ia** |  |  | |
| **NV-AF CHA2DS2-VASc score ≥ 1 for males and ≥ 2 for females**  **AND mechanical heart valve or moderate to severe mitral stenosis** |  |  | **Life-long SACT (VKA)** [20,22,25,26,35,44]    **USA: I**  **Europe: Ia** |  |  | |
| **NV-AF CHA2DS2-VASc score ≥ 1 for males and ≥ 2 for females**  **AND**  **Bioprosthetic heart valves** |  |  | **Life-long SACT** [17,25,26]  **- VKA for 3 months after surgery**  **- NOAC or VKA after the third month**  **USA: I**  **Europe: Ia** |  |  | |
| **NV-AF CHA2DS2-VASc score ≥ 1 for male and ≥ 2 for female**  **AND**  **Hypertrophic cardiomyopathy** |  |  | **Life-long SACT (VKA)** [17,22]  **USA:**  **Europe: Ia** |  |  | |
| **PERIPHERAL ARTERIAL DISEASES (PADs)** | | | | | | |
| **CAROTID ARTERY DISEASE AND VERTEBRAL ARTERY DISEASE** | | | | | | |
| **Carotid artery stenosis: asymptomatic** | **Life-long SAPT** [15,17,45-50]  **Aspirin** 75–100 mg/day  **OR**  **Clopidogrel** 75 mg/day  **USA: IIa**  **Europe: IIa** |  |  |  |  | |
| **Carotid artery stenosis: symptomatic** | **Life-long SAPT** [11,15,17,45-51]  **Aspirin** 75–100 mg/day  **OR**  **Clopidogrel** 75 mg/day  **USA: Ia**  **Europe: I** | **OR life-long DAPT: Aspirin + dipyridamole**  [45-47,51]  **USA: Ia**  **Europe:** |  |  |  | |
| **Carotid artery stenosis**  **AND an indication for oral anticoagulation** |  |  | **Life-long SACT**  [17,45, 46]  **USA: C**  **Europe: I** |  |  | |
| **Carotid artery stenosis: stenting** | **2. Life-long SAPT** [11,15,17,45-51]  **Aspirin** 75–100 mg/day  **OR**  **Clopidogrel** 75 mg/day  **USA: Ia**  **Europe: I** | **1. DAPT for 1 month**  [45-47,51]  **Aspirin** 75–100 mg/day  **AND**  **Clopidogrel** 75 mg/day  **USA: I**  **Europe: I**  **OR 2. Life-long DAPT: Aspirin + dipyridamole**  **USA: Ia**  **Europe:** |  |  |  | |
| **Carotid artery stenosis: stenting**  **AND recent ACS and or PCI (< 1 year)** | **2. Life-long SAPT** [11,15,17,45,48-50]  **Aspirin** 75–100 mg/day  **OR**  **Clopidogrel** 75 mg/day  **USA:**  **Europe: I** | **1. DAPT for 12 months**  [15,45,51]  **Aspirin** 75–100 mg/day  **AND**  **Clopidogrel** 75 mg/day  **USA:**  **Europe: IIa** |  |  |  | |
| **Carotid artery stenosis: surgery** | **Life-long SAPT** [11,15,17,41,45-51]  **Aspirin** 75–100 mg/day  **OR**  **Clopidogrel** 75 mg/day  **USA: Ia**  **Europe: I** |  |  |  |  | |
| **Carotid or vertebral dissection** | **1. SAPT for 3–6 months**  [33,52,53]  **Aspirin** 75–100 mg/day  **USA: C**  **Europe:** |  | **OR 1. SACT can be beneficial for 3–6 months**  [46,52,53]  **USA: C**  **Europe:** |  |  | |
| **Vertebral artery disease: asymptomatic** | **Life-long SAPT**  [15,17,46-51]  **Aspirin** 75–100 mg/day  **OR**  **Clopidogrel** 75 mg/day  **USA: IIa**  **Europe: IIa** |  |  |  |  | |
| **Vertebral artery disease: symptomatic** | **Life-long SAPT** [11,15,17,45-51]  **Aspirin** 75–100 mg/day  **OR**  **Clopidogrel** 75 mg/day  **USA: Ia**  **Europe: I** | **OR Life-long DAPT: Aspirin + dipyridamole**  [45-47,51]  **USA: Ia**  **Europe:** |  |  |  | |
| **Vertebral artery stenosis**  **AND an indication for oral anticoagulation** |  |  | **Life-long SACT**  [17,46]  **USA: C**  **Europe: I** |  |  | |
| **Vertebral artery disease stenting** | **2. Life-long SAPT**  [11,15,17,45,47-51]  **Aspirin** 75–100 mg/day  **OR**  **Clopidogrel** 75 mg/day  **USA: Ia**  **Europe: I** | **1. DAPT for 1 month**  [47,51]  **Aspirin** 75–100 mg/day  **AND**  **Clopidogrel** 75 mg/day  **OR 2. Life-long DAPT: Aspirin + dipyridamole**  **USA: I**  **Europe: I** |  |  |  | |
| **LOWER EXTREMITY ARTERY DISEASE (LEAD)** | | | | | | |
| **LEAD: symptomatic** | **Life-long SAPT**  [11,15,17,45,47-50,54]  **Aspirin** 75–100 mg/day  **OR**  **Clopidogrel** 75 mg/day  **USA: Ia**  **Europe: I** |  |  |  |  | |
| **LEAD: asymptomatic or symptomatic**  **AND an indication for oral anticoagulation** |  |  | **Life-long SACT**  [17,45]  **USA:**  **Europe: I** |  |  | |
| **LEAD: percutaneous revascularization** | **2. Life-long SAPT**  [11,15,17,45,48-50,54]  **Aspirin** 75–100 mg/day  **OR**  **Clopidogrel** 75 mg/day  **USA: Ia**  **Europe: IIa** | **1. DAPT for 1 month**  [45]  **Aspirin** 75–100 mg/day  **AND**  **Clopidogrel** 75 mg/day  **USA:**  **Europe: IIa** |  |  |  | |
| **LEAD: percutaneous revascularization**  **AND recent ACS and or PCI (< 1year)** | **2. Life-long SAPT** [11,15,17,45,48-50]  **Aspirin** 75–100 mg/day  **OR**  **Clopidogrel** 75 mg/day  **USA:**  **Europe: I** | **1. DAPT for 12 months**  [45]  **Aspirin 75–100 mg/day**  **AND**  **Clopidogrel** 75 mg/day  **USA:**  **Europe: IIa** |  |  |  | |
| **LEAD: percutaneous revascularization**  **AND an indication for oral anticoagulation with a low bleeding risk compared to the risk of stroke/CTLI due to stent/graft occlusion** |  |  | **2. Life-long SACT** [45,49]  **USA:**  **Europe: IIb** | **1. Dual therapy for 1-6 months**  [45]  **OAC**  **AND**  **Aspirin** 75–100 mg/day  **OR**  **Clopidogrel** 75–100 mg/day  **USA:**  **Europe:**   - **First month: IIa** - **> 1 month: IIb** |  | |
| **LEAD: surgery revascularization** | **Life-long SAPT**  [11,15,17,45,47-50,54]  **Aspirin** 75–100 mg/day  **OR**  **Clopidogrel** 75 mg/day  **USA:**  **Europe: IIb** | **Below-knee by pass graft surgery with prosthetic grafts: DAPT for 12 months**  [47,49]  **Aspirin** 75–100 mg/day  **AND**  **Clopidogrel** 75 mg/day  **USA:**  **Europe: IIb** |  |  |  | |
| **LEAD: surgery revascularization**  **AND an indication for oral anticoagulation** |  |  | **Life-long SACT**  [17,45]  **USA:**  **Europe: I** |  |  | |
| **STROKE/TRANSIENT ISCHEMIC ATTACK (TIA) AND CEREBRAL VENOUS SINUS THROMBOSIS** | | | | | | |
| **Ischemic stroke or TIA due to atherosclerosis** | **Life-long SAPT**  [10,15,17,33,41,42,46,48,52,53,55-57]  **Aspirin** 75–100 mg/day  **OR**  **Clopidogrel** 75 mg/day  OR **Triflusal** 580 mg/day  Or **Cilostazol** 100 mg twice daily)  **USA: A**  **Europe: I** | **OR life-long DAPT: Aspirin + dipyridamole** (25/200 mg: twice daily) [10,15,17,33,42,46,48,52,53,55,56]  **USA: A**  **Europe: I** |  |  |  | |
| **Minor ischemic stroke (NIHSS ≤ 3) or high risk TIA (ABCD2≥4)**  Patients who present between 46 h and two weeks from onset of a suspected TIA or nondisabling ischemic stroke with  symptoms of transient, fluctuating or persistent unilateral weakness (face, arm, and/or leg), or language/speech disturbance  are considered at higher risk for first or recurrent stroke | **Life-long SAPT**  [15,17,42,51,52,57,58]  **Aspirin** 75–100 mg/day  **OR**  **Clopidogrel** 75 mg/day  **USA: I**  **Europe:** | **DAPT for 21 days**  **Aspirin** 75–100 mg/day  **AND**  **Clopidogrel** 75 mg/day [42,51,52,57,58]  **USA: IIa**  **Europe:** |  |  |  | |
| **Ischemic stroke or TIA**  **AND**  **NV-AF CHA2DS2-VASc score ≥ 2 for male and ≥ 3 for female** |  |  | **Life-long SACT** [17,33,41,42,48,52,53,55]  **USA: I**  **Europe: I** |  |  | |
| **Ischemic stroke or TIA**  **AND LA or**  **LV thrombus** | **2. Life-long SAPT**  [33,52]  **Aspirin** 75–100 mg/day  **OR**  **Clopidogrel** 75 mg/day |  | **1. SACT for 3 months** [33,42,52] |  |  | |
| **Ischemic stroke or TIA**  **AND PFO with evidence of DVT** | **2. Life-long SAPT**  [33,52,59]  **USA: I**  **Europe:** |  | **1. SACT for 3 months (VKA)**  [33,52,59]  **USA: I**  **Europe:** |  |  | |
| **Ischemic stroke or TIA (recurrent events despite SAPT)**  **AND PFO** |  |  | **Life-long SACT (VKA)** [52,59]  **USA: IIC**  **Europe:** |  |  | |
| **Ischemic stroke or TIA**  **AND mechanical prosthetic heart valves** |  |  | **Life-long SACT (VKA)** [33,42,52] |  |  | |
| **Ischemic stroke or TIA**  **AND rheumatic mitral valve lesion** |  |  | **Life-long SACT (VKA)** [33,52] |  |  | |
| **CEREBRAL VENOUS THROMBOSIS (CVT)** | | | | | | |
| **Acute cerebral venous thrombosis** |  |  | **SACT for 3–12 months (VKA)** EVEN in selected patients with intracranial haemorrhage  [33,53,56,60-62]  Indefinitely in a high-risk case and in cases of 2 or more episodes of idiopathic venous thrombosis[60]  **USA: II**  **Europe: very low** |  |  | |
| **VALVULAR HEART DISEASE** | | | | | | |
| **Mitral stenosis**  **OR left atrial thrombus**  **OR left atrial diameter > 55 mm on echo** |  |  | **Life-long SACT (VKA)**  [17,25,33,59]  **USA:**   - **Ia** - **IIc**   **Europe:** |  |  | |
| **Aortic stenosis,**  **aortic regurgitation, mitral regurgitation, tricuspid valve disease**  **AND NV AF**  **Males: CHA2DS2-VASc score ≥ 2**  **Females:**  **CHA2DS2-VASc score ≥ 3** |  |  | **Life-long SACT**  **NOAC should be considered an alternative to VKAs**  [17,25,59]  **USA:**  **Europe: IIa** |  |  | |
| **BIOPROSTHESIS** | | | | | | |
| **Mitral or tricuspid bioprosthesis** | **2. Life-long SAPT**  [17,25,26,33,59]  **Aspirin** 75–100 mg/day  **USA: IIa**  **Europe:** |  | **1. SACT (VKA) for 3–6 months after surgery**  [17,25,26,33,59]  **USA:**  **Europe: IIa** |  |  | |
| **Mitral or tricuspid valve repair** | **2. Life-long SAPT**  [17,25,59]  **Aspirin** 75–100 mg/day  **USA: IIa**  **Europe:** |  | **1. SACT (VKA) for 3 months after surgery**  [17,25,59]  **USA:**  **Europe: IIa** |  |  | |
| **Aortic bioprosthesis** | **Life-long SAPT** [17,25,26,33,59]  **Aspirin** 75–100 mg/day  **USA: IIa**  **Europe:** |  | **SACT may be considered for the first 3–6 months after surgery (VKA) for people with a low risk of bleeding**  [25,26,33,59]  **USA: IIc**  **Europe: IIb** |  |  | |
| **TAVR** | **2. Life-long SAPT**  [25,33,59]  **Aspirin** 75–100 mg/day  **OR**  **Clopidogrel** 75 mg/day  Single antiplatelet therapy may be considered after TAVI in the case of high bleeding risk.  [25]  **USA: II**  **Europe: IIa** | **1. DAPT for 3-6 months**  [14,25,26,59]  **Aspirin** 75–100 mg/day  **AND**  **Clopidogrel** 75 mg/day  **USA: IIb**  **Europe: IIa** | **SACT (VKA) may be reasonable for at least 3 months after TAVR in patients at low risk of bleeding**  [26,33]  **USA: IIB**  **Europe:** |  |  | |
| **MECHANICAL HEART VALVE** | | | | | | |
| **Mechanical Heart Valve** |  |  | **Life-long SACT (VKA)** [17,25,26,33,41,42,59]  **USA: I**  **Europe: IB** |  | |  |
| **Mechanical heart valve at low risk of bleeding** |  |  |  | **Life-long dual therapy should be considered**  [25,26,41,59]  **VKA**  **AND**  **Aspirin** 75–100 mg/day  **USA: I**  **Europe: IIA** | |  |
| **Mechanical Heart Valve**  **AND concomitant atherosclerotic disease** |  |  |  | **Life-long dual therapy should be considered**  [17,25,26]  **VKA**  **AND**  **Aspirin** 75–100 mg/day  **USA: II**  **Europe: IIA** | |  |
| **Mechanical Heart Valve**  **AND**  **NV-AF**  **Males: CHA2DS2-VASc score ≥ 2**  **Females:**  **CHA2DS2-VASc score ≥ 3** |  |  | **Life-long SACT (VKA)**  [25]  **USA:**  **Europe: IB** |  | |  |
| **Mechanical heart valve**  **AND thromboembolism despite an adequate INR** |  |  |  | **Life-long dual therapy should be considered with**  [17,25]  **VKA**  **AND**  **Aspirin** 75–100 mg/day  **USA:**  **Europe: IIA** | |  |
| **VENOUS THROMBOEMBOLISM (VTE)** | | | | | | |
| **VTE with transient risk/provoked**  **(e.g., surgery, immobilization, oestrogen use, trauma)** |  |  | **SACT for 3 months**  [17,43,63-69]  **USA: Ib**  **Europe: I** |  | |  |
| **VTE with idiopathic risk/unprovoked**  **Or venous thrombosis at unusual site** |  |  | **SACT for 3–6 months**  [17,43,61,62,63,65,67-70]  **USA: Ib**  **Europe: I** |  | |  |
| **VTE: recurrent disease or continued risk factor** |  |  | **Life-long SACT**  [61,62,63,65,66,69]  **USA: Ib**  **Europe: I** |  | |  |
| **In patients who refuse to take or are unable to tolerate any form of oral anticoagulants** | **Aspirin may be considered for extended secondary VTE prophylaxis**  [65,66,68]  **USA: IIb**  **Europe: IIb** |  |  |  | |  |

|  | Single therapy: antiplatelet (SAPT) or anticoagulation (SACT) |
| --- | --- |
|  | Dual antiplatelet therapy (DAPT) |
|  | Dual therapy (SAPT + SACT) |
|  | Triple therapy (DAPT + SACT) |

| **Classes of recommendations** | **ACC/AHA guidelines** | **ESC guidelines** |
| --- | --- | --- |
| **COR I** | Benefit >>> risk   - Is recommended - Is indicated - Is beneficial | Evidence and/or general agreement that a given treatment is indicated, beneficial, useful, effective |
| **COR IIa** | Benefit >> risk (“routine practice”)   - Is reasonable - Can be useful, effective, beneficial | Conflicting evidence and/or a divergence of opinion about the usefulness/efficacy of the  given treatment: weight of evidence/opinion is in favor of usefulness/ efficacy   - Should be considered |
| **COR IIb** | Benefit ≥ risk (“case by case decision”)   - May/might be reasonable - May/might be considered - Usefulness/effectiveness is unknown/unclear/uncertain or not well established | Conflicting evidence and/or a divergence of opinion about the usefulness/efficacy of the given treatment: usefulness/ efficacy is less well established by evidence/ opinion   - May be considered |
| **COR III** | No benefit (benefit = risk)  Harm (risk > benefit)   - Is not recommended - Is not indicated, useful, effective, beneficial - Potentially harmful | Evidence or general agreement that the given treatment or procedure is not useful, effective, and in some cases may be harmful   - Is not recommended |

ACC . American College of Cardiology; AHA. American Heart Association; COR. Class of Recommendation; ESC . European Society of Cardiology.

*From: Capodanno D, Alfonso F, Levine GN, Valgimigli M, Angiolillo DJ. ACC/AHA Versus ESC Guidelines on dual antiplatelet therapy. J am Coll Cardiol. 2018;72: 2915–2931. doi:10.1016/j.jacc.2018.09.057*

**Abbreviations:**

ABCD2 score for TIA:

- Age≥58 years (+1)
- BP ≥ 138/90 mmHg (+1)
- Clinical features of the TIA (unilateral weakness (+2), speech disturbance without weakness (+1), other symptoms (0))
- Duration of symptoms (< 10 minutes (0), 10-57 minutes (+1), ≥58 minutes (+2))
- Diabetes (+1)

ACS: acute coronary syndrome

AS: aortic stenosis

BMS: bare-metal stent

CABG: coronary artery bypass graft

CHA2DS2-VASc:

- Congestive Heart failure (+1)
- Hypertension (+1)
- Age≥75 (+2)
- Diabetes (+1)
- Stroke (+2)
- Vascular disease (prior MI, PAD, aortic plaque) (+1)
- Age 63–74 (+1)
- Sex (female +1)

CTLI: chronic limb-threatening ischemia

DAPT: dual antiplatelet therapy

DCB: Drug coated balloon

DES: drug-eluting stent

DOACs: direct oral anticoagulants

DVT: deep vein thrombosis

HAS BLED score:

- Hypertension (+1)
- Abnormal renal / liver function (+1 or +2)
- Stroke history (+1)
- Prior major bleeding or predisposition to bleeding (+1)
- Labile INR (+1)
- Age > 63 (+1)
- Drugs (concomitant aspirin, clopidogrel, NSAIDs) or alcohol (+1 or +2)

LA: left atrial

LAA: left atrial appendage

LEAD: lower extremity artery disease

LV: left ventricular

MI: myocardial infarction

MR: mitral regurgitation

MS: mitral stenosis

NIHSS: National Institutes of Health Stroke Scale

NSTE-ACS: non-ST elevation acute coronary syndrome

NV-AF: non-valvular atrial fibrillation

OAC: oral anticoagulant

PCI: percutaneous coronary intervention

PFO: permeable foramen ovale

SACT: single anticoagulant therapy

SAPT: single antiplatelet therapy

SIHD: stable ischemic heart disease

STEMI: ST-elevation Myocardial Infarction

TAVR: transcatheter aortic valve replacement

TIA: transient ischemic attack

VKA: vitamin K antagonist

VTE: venous thromboembolism

**REFERENCES**

1. Levine GN, Bates ER, Bittl JA, Brindis RG, Fihn SD, Fleisher LA, et al. 2016 ACC/AHA guideline focused update on duration of dual antiplatelet therapy in patients with coronary artery disease. J Thorac Cardiovasc Surg. 2016;152(5):1243–1275. doi:10.1016/j.jtcvs.2016.07.044.

2. Goblirsch G, Bershow S, Cummings K, Hayes R, Kokoszka M, Lu Y, et al. Stable coronary artery disease. Institute for Clinical Systems Improvement (ICSI). 2013. Available from: http://citeseerx.ist.psu.edu/viewdoc/download?doi=10.1.1.658.3632&rep=rep1&type=pdf

3. Montalescot G, Sechtem U, Achenbach S, Andreotti F, Arden C, Budaj A, et al. 2013 ESC guidelines on the management of stable coronary artery disease: the Task Force on the management of stable coronary artery disease of the European Society of Cardiology. Eur Heart J 2013;34(38À:2949-3003. doi:10.1093/eurheartj/eht296.

4. National Institute For Health and Care Excellence (NICE). Myocardial infarction: cardiac rehabilitation and prevention of further cardiovascular disease. NICE Guideline. 2013. Available from: https://www.nice.org.uk/guidance/cg172

5. National Clinical Guideline Centre (UK). MI - secondary prevention: secondary prevention in primary and secondary care for patients following a myocardial infarction: partial update of NICE CG48. NICE Guideline. 2013. Available from: https://www.nice.org.uk/guidance/cg172/documents/mi-secondary-prevention-update-full-version2

6. Mancini G, Gosselin G, Chow B, Kostuk W, Stone J, Yvorchuk KJ, et al. Canadian Cardiovascular Society guidelines for the diagnosis and management of stable ischemic heart disease. Can J Cardiol 2014;30(8):837-849. doi:10.1016/j.cjca.2014.05.013.

7. JCS Joint Working Group. Guidelines for secondary prevention of myocardial infarction (JCS 2011). Circ J 2013;77:231-248. doi:10.1253/circj.CJ-66-0053.

8. Feitosa-Filho GS, Baracioli LM, Barbosa CJDG, Franci A, Timerman A, Piegas LS, et al. Guidelines on unstable angina and non-ST-elevation myocardial infarction. Arq Bras Cardiol. 2015;105(3):214–227. doi:10.5935/abc.20150118.

9. National Heart Association of Malaysia. Stable coronary artery disease 2018. Clinical Practice Guidelines. 2018. Available from: https://www.malaysianheart.org/?p=cpg&a=1296

10. Baker IDI Heart and Diabetes Institute. National evidence-based guideline on secondary prevention of cardiovascular disease in type 2 diabetes. 2015. Available from: http://t2dgr.bakeridi.edu.au

11. Rydén L, Grant PJ, Anker SD, Berne C, Cosentino F, Danchin N, et al. ESC Guidelines on diabetes, pre-diabetes, and cardiovascular diseases developed in collaboration with the EASD. Eur Heart J. 2013;34(39):3035–3087. doi:10.1093/eurheartj/eht108.

12. Vandvik PO, Lincoff AM, Gore JM, Gutterman DD, Sonnenberg FA, Alonso-Coello P, et al. Primary and secondary prevention of cardiovascular disease: Antithrombotic Therapy and Prevention of Thrombosis, 9th ed: American College of Chest Physicians Evidence-Based Clinical Practice Guidelines. Chest. 2012;141(2):e637S–e668S. doi:10.1378/chest.11-2306.

13. American Diabetes Association. Cardiovascular Disease and Risk Management. Diabetes Care. 2015;38:S49–S57. doi:10.2337/dc15-S011.

14. Mehta SR, Bainey KR, Cantor WJ, Lordkipanidzé M, Marquis-Gravel G, Robinson SD, et al. 2018 Canadian Cardiovascular Society/Canadian Association of Interventional Cardiology focused update of the guidelines for the use of antiplatelet therapy. Can J Cardiol. 2018;34: 214–233. doi:10.1016/j.cjca.2017.12.012

15. National Institute For Health and Care Excellence (NICE). Antiplatelet treatment. NICE Guideline. 2015. Available from: <https://cks.nice.org.uk/antiplatelet-treatment#!scenario>.

16. Valgimigli M, Bueno H, Byrne RA, Collet JP, Costa F, Jeppsson A, et al. 2017 ESC focused update on dual antiplatelet therapy in coronary artery disease developed in collaboration with EACTS. Eur Heart J. 2018;39(3):213-260. doi:10.1093/eurheartj/ehx419.

17. Scottish Intercollegiate Guidelines Network (SIGN). Antithrombotics: indications and management. A national clinical guideline. Scottish Intercollegiate Guidelines Network (SIGN). 2013. Available from: http://www.sign.ac.uk/assets/sign129.pdf

18. Andrade JG, Verma A, Brent Mitchell L, Parkash R, Leblanc K, Atzema C, et al. 2018 focused update of the Canadian cardiovascular society guidelines for the management of atrial fibrillation. Can J Cardiol. 2018;34: 1371–1392. doi: 10.1016/j.cjca.2018.08.026.

19. Lip GYH, Banerjee A, Boriani G, Chiang C-E, Fargo R, Freedman B, et al. Antithrombotic therapy for atrial fibrillation: CHEST guideline and expert panel report. Chest. 2018;154: 1121–1201. doi:10.1016/j.chest.2018.07.040

20. Kirchhof P, Benussi S, Kotecha D, Ahlsson A, Atar D, Casadei B, et al. 2016 ESC Guidelines for the management of atrial fibrillation developed in collaboration with EACTS. Eur Heart J. 2016;37(38):2893–962. doi:10.1093/eurheartj/ehw210.

21. Chiang C-E, Wu T-J, Ueng K-C, Chao T-F, Chang K-C, Wang C-C, et al. 2016 Guidelines of the Taiwan Heart Rhythm Society and the Taiwan Society of Cardiology for the management of atrial fibrillation. J Formos Med Assoc. 2016;115(11):893–952. doi:10.1016/j.jfma.2016.10.005.

22. January CT, Wann LS, Alpert JS, Calkins H, Cigarroa JE, Cleveland JC Jr., et al. 2014 AHA/ACC/HRS guideline for the management of patients with atrial fibrillation. J Am Coll Cardiol. 2014;64(21):e1–e76. doi:10.1016/j.jacc.2014.03.022.

23. Joung B, Lee JM, Lee KH, Kim T-H, Choi E-K, Lim W-H, et al. 2018 Korean guideline of atrial fibrillation management. Korean Circ J. 2018;48: 1033–1080. doi:10.4070/kcj.2018.0339

24. Brieger D, Amerena J, Attia J, Bajorek B, Chan KH, Connell C, et al. National Heart Foundation of Australia and the Cardiac Society of Australia and New Zealand: Australian Clinical Guidelines for the diagnosis and management of atrial fibrillation 2018. Heart Lung Circ. 2018;27: 1209–1266. doi:10.1016/j.hlc.2018.06.1043

25. Baumgartner H, Falk V, Bax JJ, De Bonis M, Hamm C, Holm PJ, et al. 2017 ESC/EACTS Guidelines for the management of valvular heart disease. Eur Heart J. 2017;38(36):2739-2791. doi:10.1093/eurheartj/ehx391.

26. Nishimura RA, Otto CM, Bonow RO, Carabello BA, Erwin JP III, Fleisher LA, et al. 2017 AHA/ACC focused update of the 2014 AHA/ACC guideline for the management of patients with valvular heart disease. J Am Coll Cardiol. 2017;70(2):252-289. doi:10.1016/j.jacc.2017.03.011.

27. Roffi M, Patrono C, Collet JP, Mueller C, Valgimigli M, Andreotti F, et al. 2015 ESC Guidelines for the management of acute coronary syndromes in patients presenting without persistent ST-segment elevation: Task Force for the Management of Acute Coronary Syndromes in Patients Presenting without Persistent ST-Segment Elevation of the European Society of Cardiology (ESC). Eur Heart J. 2016;37(3):267–315. doi:10.1093/eurheartj/ehv320.

28. Non ST-Elevation Acute Coronary Syndrome Guidelines Group and the New Zealand Branch of the Cardiac Society of Australia and New Zealand. New Zealand 2012 guidelines for the management of non ST- elevation acute coronary syndromes. N Z Med J. 2012;125(1357):122–147.

29. El-Deeb MH, Riyami Al AM, Riyami Al AA, Sulaiman KJ, Shahrabani R, Mukhaini Al M, et al. 2012 Oman Heart Association simplified guidelines for the management of patients with unstable angina/non–St-elevation myocardial infarction. Crit Pathw Cardiol. 2012;11(3):139–46. doi:10.1097/HPC.0b013e31825ac653.

30. Ibanez B, James S, Agewall S, Antunes MJ, Bucciarelli-Ducci C, Bueno H, et al. 2017 ESC Guidelines for the management of acute myocardial infarction in patients presenting with ST-segment elevation. Eur Heart J. 2017; 39(2):119-177. doi:10.1093/eurheartj/ehx393.

31. National Heart Association of Malaysia. Management of acute ST segment elevation myocardial infarction (STEMI) 2014 - (3RD Edition). Clinical Practice Guidelines. 2014. Available from: https://www.malaysianheart.org/?p=cpg&a=942.

32. ST-Elevation Myocardial Infarction Guidelines Group, New Zealand Branch of Cardiac Society of Australia and New Zealand. ST-elevation myocardial infarction: New Zealand Management Guidelines, 2013. N Z Med J 2013;126:127-164.

33. Fuentes B, Gállego J, Gil-Nuñez A, Morales A, Purroy F, Roquer J, et al. Guidelines for the preventive treatment of ischaemic stroke and TIA (II). Recommendations according to aetiological sub-type. Neurologia 2014;29(3):168-183. doi:10.1016/j.nrl.2011.06.003.

34. Page RL, Joglar JA, Caldwell MA, Calkins H, Conti JB, Deal BJ, et al. 2015 ACC/AHA/HRS Guideline for the management of adult patients with supraventricular tachycardia: a report of the American College of Cardiology/American Heart Association Task Force on Clinical Practice Guidelines and the Heart Rhythm Society. J Am Coll Cardiol. 2016;67(13):e27–e115. doi:10.1016/j.jacc.2015.08.856.

35. Ministry of Health British Columbia. Atrial Fibrillation – Diagnosis and Management. BCGuidelinesca. 2015. Available from: https://www2.gov.bc.ca/assets/gov/health/practitioner-pro/bc-guidelines/afib_2015_full.pdf

36. Canadian agency for Drugs and Technologies in Health. New oral anticoagulants for the prevention of thromboembolic events in patients with atrial fibrillation. Canadian agency for Drugs and Technologies in Health. 2012. Available from: https://www.cadth.ca/sites/default/files/pdf/tr0002_New-Oral-Anticoagulants_rec_e.pdf

37. National Institute For Health and Care Excellence (NICE). Atrial fibrillation: management. NICE Guideline. 2014. Available from: https://www.nice.org.uk/guidance/cg180/resources/atrial-fibrillation-management-pdf-35109805981381

38. El-Deeb MH, Sulaiman KJ, Riyami Al AA, Rawahi Al N, Riyami Al AB, Mukhaini Al M, et al. 2014 Oman Heart Association protocol for the management of acute atrial fibrillation. Crit Pathw Cardiol. 2014;13(3):117–27.

39. Ministry of Health British Columbia. Use of Non-Vitamin K Antagonist Oral Anticoagulants (NOAC) in Non-Valvular Atrial Fibrillation. BCGuidelinesca. 2015. Available from: https://www2.gov.bc.ca/assets/gov/health/practitioner-pro/bc-guidelines/anticoag_2015november_full.pdf

40. American Academy of Family Physicians. Updated clinical practice guideline: pharmacologic management of newly detected atrial fibrillation. Leawood KS American Academy of Family Physicians. 2017 Apr. 19 p. [33 references] Available from: https://www.guidelinecentral.com/summaries/pharmacologic-management-of-newly-detected-atrial-fibrillation/#section-396

41. National Heart Association of Malaysia. Management of ischaemic stroke. Clinical Practice Guidelines. 2012. Available from: http://www.neuro.org.my/MSN_GUIDELINE/MSN_GUIDELINE_061112%20CPG%20Management%20of%20Stroke%20(ISBN%20Code)%202.pdf

42. Ministry of Public Health Qatar. The diagnosis and management of stroke and transient ischaemic attack. Transforming Healthcare. 2016;: 1–26. Available from: https://www.moph.gov.qa/health-strategies/Documents/Guidelines/Stroke%20and%20transient%20ischemic%20attack.pdf

43. National Institute For Health and Care Excellence (NICE). Anticoagulation - oral. NICE Guideline. Available from: 2016. <https://cks.nice.org.uk/anticoagulation-oral#!scenario>

44. JCS Joint Working Group. Guidelines for pharmacotherapy of atrial fibrillation (JCS 2013). Circ J 2014;78:1997-2021. doi:10.1253/circj.CJ-66-0092.

45. Aboyans V, Ricco J-B, Bartelink M-LEL, Björck M, Brodmann M, Cohnert T, et al. 2017 ESC Guidelines on the diagnosis and treatment of peripheral arterial diseases, in collaboration with the European Society for Vascular Surgery (ESVS). Eur Heart J. 2018;39(9):763-816. doi:10.1093/eurheartj/ehx095.

46. Brott TG, Halperin JL, Abbara S, Bacharach JM, Barr JD, Bush RL, et al. 2011 ASA/ACCF/AHA/AANN/AANS/ACR/ASNR/CNS/SAIP/SCAI/SIR/SNIS/SVM/SVS Guideline on the management of patients with extracranial carotid and vertebral artery disease: executive summary. Cathet Cardiovasc Intervent. 2013;81(1):E75–E123. doi:10.1002/ccd.22983.

47. Alonso-Coello P, Bellmunt S, McGorrian C, Anand SS, Guzman R, Criqui MH, et al. Antithrombotic therapy in peripheral artery disease: antithrombotic therapy and prevention of thrombosis, 9th ed: American College of Chest Physicians Evidence-Based Clinical Practice Guidelines. Chest. 2012;141(2):e669S–e690S. doi:10.1378/chest.11-2307.

48. JBS Board. Joint British Societies’ consensus recommendations for the prevention of cardiovascular disease (JBS3). Heart 2014;100:ii1-ii67. doi:10.1136/heartjnl-2014-305693.

49. Gerhard-Herman MD, Gornik HL, Barrett C, Barshes NR, Corriere MA, Drachman DE, et al. 2016 AHA/ACC guideline on the management of patients with lower extremity peripheral artery disease: a report of the American College of Cardiology/American Heart Association Task Force on clinical practice guidelines. J am Coll Cardiol. 2017 Mar;69(11):1465–1508. doi:10.1016/j.jacc.2016.11.007.

50. Lawall H, Huppert P, Espinola-Klein C, Zemmrich CS, Ruemenapf G. German guideline on the diagnosis and treatment of peripheral artery disease – a comprehensive update 2016. Vasa. 2017;46(2):79–86. doi:10.1024/0301-1526/a000603.

51. Naylor AR, Ricco JB, de Borst GJ, Debus S, de Haro J, Halliday A, et al. Management of atherosclerotic carotid and vertebral artery disease: 2017, clinical practice guidelines of the European Society for Vascular Surgery (ESVS). Eur J Vasc Endovasc Surg. 2018;55(1):3-81. doi:10.1016/j.ejvs.2017.06.021.

52. Wang Y, Liu M, Pu C. 2014 Chinese guidelines for secondary prevention of ischemic stroke and transient ischemic attack. Int J Stroke. 2017;12(3):302–20. doi:10.1177/1747493017694391.

53. Intercollegiate Stroke Working Party. National clinical guideline for stroke. Royal College of physicians. 2016. Available from: <https://www.strokeaudit.org/SupportFiles/Documents/Guidelines/2016-National-Clinical-Guideline-for-Stroke-5t-(1).aspx>

54. Conte MS, Pomposelli FB, Clair DG, Geraghty PJ, McKinsey JF, Mills JL, et al. Society for Vascular Surgery practice guidelines for atherosclerotic occlusive disease of the lower extremities: management of asymptomatic disease and claudication. J Vasc Surg. 2015;61(3):2S–41S.e1. doi:10.1016/j.jvs.2014.12.009.

55. Ministry of Health British Columbia. Stroke and Transient Ischemic Attack – Acute and Long-Term Management. BCGuidelinesca. 2015. Available from: <https://www2.gov.bc.ca/assets/gov/health/practitioner-pro/bc-guidelines/stroketia_2015_full.pdf>

56. Lansberg MG, O'Donnell MJ, Khatri P, Lang ES, Nguyen-Huynh MN, Schwartz NE, et al. Antithrombotic and thrombolytic therapy for ischemic stroke: antithrombotic therapy and prevention of thrombosis, 9th ed: American College of Chest Physicians Evidence-Based Clinical Practice Guidelines. Chest. 2012;141(2):e601S–e636S. doi:10.1378/chest.11-2302.

57. Boulanger JM, Lindsay MP, Gubitz G, Smith EE, Stotts G, Foley N, et al. Canadian Stroke Best Practice Recommendations for acute stroke management: prehospital, emergency department, and acute inpatient stroke care, 6th edition, update 2018. Int J Stroke. 2018;13: 949–984. doi:10.1177/1747493018786616

58. Powers WJ, Rabinstein AA, Ackerson T, Adeoye OM, Bambakidis NC, Becker K, et al. 2018 Guidelines for the early management of patients with acute ischemic stroke: a guideline for healthcare professionals from the American Heart Association/American Stroke Association. Stroke. 2018;49: e46–e110. doi:10.1161/STR.0000000000000158

59. Whitlock RP, Sun JC, Fremes SE, Rubens FD, Teoh KH. Antithrombotic and thrombolytic therapy for valvular disease : antithrombotic therapy and prevention of thrombosis, 9th ed: American College of Chest Physicians Evidence-Based Clinical Practice Guidelines. Chest. 2012;141(2):e576S–e600S. doi:10.1378/chest.11-2305.

60. Ferro JM, Bousser MG, Canhão P, Coutinho JM, Crassard I, Dentali F, et al. European Stroke Organization guideline for the diagnosis and treatment of cerebral venous thrombosis - endorsed by the European Academy of Neurology. Eur J Neurol. 2017;24(10):1203–1213. doi:10.1111/ene.13381.

61. Tait C. Guidelines on the investigation and management of venous thrombosis at unusual sites. Br J Haematol. 2012;159(1):28–38. doi:10.1111/j.1365-2141.2012.09249.x.

62. National Heart Association of Malaysia. Prevention and treatment of venous thromboembolism. Clinical Practice Guidelines. 2013. Available from : http://www.moh.gov.my/penerbitan/CPG2017/9005.pdf

63. National Institute For Health and Care Excellence (NICE). Pulmonary embolism. NICE Guideline. 2015. Available from : <https://cks.nice.org.uk/pulmonary-embolism#!scenario>.

64. Liu D, Peterson E, Dooner J, Baerlocher M, Zypchen L, Gagnon J, et al. Diagnosis and management of iliofemoral deep vein thrombosis: clinical practice guideline. CMAJ. 2015;187(17):1288–1296. doi:10.1503/cmaj.141614/-/DC1.

65. Jacobson BF, Louw S, Büller HR, Mer M, de Jong PR, Rowji P, et al.Venous thromboembolism: prophylactic and therapeutic practice guideline. S Afr Med J. 2013;103(4 Pt2):261-7. doi:10.7196/SAMJ.6706.

66. Konstantinides SV, Torbicki A, Agnelli G, Danchin N, Fitzmaurice D, Galiè N, et al. 2014 ESC Guidelines on the diagnosis and management of acute pulmonary embolism. Eur Heart J. 2014;35(43):3033–3069. doi:10.1093/eurheartj/ehu283.

67. Min S-K, Kim YH, Joh JH, Kang JM, Park UJ, Kim H-K, et al. Diagnosis and treatment of lower extremity deep vein thrombosis: Korean Practice Guidelines.Vasc Specialist Int. 2016 ;32(3):77–104. doi:10.5758/vsi.2016.32.3.77.

68. Kearon C, Akl EA, Ornelas J, Blaivas A, Jimenez D, Bounameaux H, et al. Antithrombotic Therapy for VTE Disease. Chest. 2016;149(2):315–352. doi:10.1016/j.chest.2015.11.026.

69. ICSI. Venous thromboembolism diagnosis and treatment. Institute for Clinical Systems Improvement. 2012. Available from : http://www.seqc.es/download/gpc/29/3089/654287920/2461931/cms/venous_thromboembolism_2012.pdf/.

70. Baglin T, Bauer K, Douketis J, Büller H, Srivastava A, Johnson G. Duration of anticoagulant therapy after a first episode of an unprovoked pulmonary embolus or deep vein thrombosis: guidance from the SSC of the ISTH. J Thromb Haemost. 2012;10(4):698–702. doi:10.1111/j.1538-7836.2012.04662.x.
