# Supplementary material for: A prescription support-tool for chronic management of oral antithrombotic combinations in adults based on a systematic review of international guidelines
Source: PLoS One. 2019 Feb 14;14(2):e0211695. doi: 10.1371/journal.pone.0211695 (PMC6375571; doi:10.1371/journal.pone.0211695)
Supplement: S2 Table — (DOCX) [file pone.0211695.s005.docx]

**S2 Table: Specificities from guidelines about older people**

| **Reference** | **Recommendations** | **Grade** |
| --- | --- | --- |
| **Joung et al, 2018**  **About atrial fibrillation**  [1] | The elderly population is fragile and prone to falls. Nonetheless, OAC use is recommended in elderly AF patients because of the high benefit/risk ratio. Thus, OACs may still be considered as thromboprophylaxis for elderly patients, with NOACs being the more favorable choice. The Korean AF guideline recommends low-dose rivaroxaban (15 mg q.d.) in patients aged ≥80 years. | **No grade** |
| **Brieger et al, 2018**  **About atrial fibrillation**  [2] | Practice advice: The combination of impaired hepatic, renal, cognitive function, high-risk medications, and poly-pharmacy make drug interactions and complications more likely. Furthermore, these patients are usually excluded from randomised trials and observational registries, so there are few data to guide decisions. An integrated management approach would be of particular value in this cohort (see Section 7). | **No grade** |
| **Ministry of health, Malaysia, 2018**  [3]  **About stable coronary artery disease** | Management should be individualised taking into consideration comorbidities and should not be based on age alone. The choice of revascularization in the elderly should be discussed by the Heart Team considering local expertise and patient preferences. | **No grade** |
| **Ibanez et al, 2017**  [4]  **About ST-elevation Myocardial Infarction** | “It is key to maintain a high index of suspicion for myocardial infarction in elderly patients who present with atypical complaints, treating them as recommended, and using specific strategies to reduce bleeding risk; these include paying attention to proper dosing of antithrombotic therapies, particularly in relation to renal function, frailty, or comorbidities, and using radial access whenever possible. There is no upper age limit with respect to reperfusion, especially with primary percutaneous coronary intervention”. | **No grade** |
| **Baumgartner et al, 2017**  [5]  **About valvular heart disease** | “Although current data favour transcatheter aortic valve replacement in elderly patients who are at increased risk for surgery, particularly when a transfemoral access is possible, the Heart Team should make the decision between transcatheter aortic valve replacement and Surgical aortic valve replacement after careful, comprehensive evaluation of the patient, weighing individually the risks and benefits”. | **IB** |
| **Roffi et al, 2016**  [6]  **About Non-ST Elevation Acute Coronary Syndrome** | - “It is recommended to tailor antithrombotic treatment according to bodyweight and renal function - Elderly patients should be considered for an invasive strategy and, if appropriate, revascularization after careful evaluation of potential risks and benefits, estimated life expectancy, comorbidities, quality of life, frailty and patient values and preferences”. | **IC**  **IIa** |
| **Kirchhof et al, 2016**  [7]  **About non-valvular atrial fibrillation** | - “Integrated non-valvular atrial fibrillation management and careful adaptation of drug dosing seem reasonable to reduce the complications of non-valvular atrial fibrillation therapy in such patients”. | **No grade** |
| **Page et al, 2015**  [8]  **About supraventricular tachycardia** | “Diagnostic and therapeutic approaches to supraventricular tachycardia should be individualized in patients more than 75 years of age to incorporate age, comorbid illness, physical and cognitive functions, patient preferences, and severity of symptoms”. | **IB** |
| **Baker, 2015**  [9]  **About cardiovascular disease** | “Caution should be exercised in implementing aggressive therapy in the elderly, and in those with multiple co-morbidities. These individuals are not well represented in most trials, often have a higher risk of adverse events, and their risk-benefit ratios for interventions may therefore differ from those reported in trials”. | **No grade** |
| **January et al, 2014**  [10]  **About non-valvular atrial fibrillation** | “For the older patient with non-valvular atrial fibrillation, symptoms may be minimal and somewhat atypical. The risk of stroke is increased in the elderly. For this reason, the CHA2DS2-VASc risk scoring system identifies 65 to 74 years of age as a minor risk factor for stroke and 75 years of age as a major risk factor for stroke”. | **No grade** |
| **Ministry of health, Malaysia 2014**  [11]  **About ST-elevation Myocardial Infarction** | “In the management of ST-elevation Myocardial Infarction in the elderly:   - Primary percutaneous coronary intervention - this is the preferred reperfusion strategy if facilities are available and the patient is eligible for percutaneous coronary intervention. Procedural success is highly variable. Elderly patients are more likely to have PCI related complications especially bleeding. - Fibrinolytic therapy - there is an increased risk of intracranial hemorrhage in the elderly and the risks of bleeding have to be carefully considered in those older than 75 years. - Aspirin - at a dose of 75 to 150 mg in the absence of contraindications - Clopidogrel - the absolute benefits of clopidogrel are similar, but relative benefits are less in the elderly. Patients undergoing PCI with higher TIMI risk scores or prior revascularization are more likely to benefit. A loading dose when compared to a conventional dose of clopidogrel did not result in an increased bleeding risk in the elderly. The need for a loading dose needs to be individualized. - Ticagrelor – ticagrelor has a similar efficacy as clopidogrel in patients aged ≥ 75 years of age and those < 75 years of age. There was no increased risk of bleeding. - Prasugrel - should be avoided in patients > 75 years of age   Risk stratification - this has to be individualized and patient preferences are important in determining further management. The presence of ongoing ischaemia, symptomatic malignant arrhythmias and a depressed left ventricular function are poor prognostic indicators and would generally necessitate a more aggressive approach. Experienced operators can carry out both percutaneous coronary intervention and Coronary Artery By Pass Graft, when indicated, in the elderly with acceptable morbidity and mortality. The risks are however higher than in younger patients”. | - **IIa** - **IIa** - **IIa** - **IIa** - **IIa** - **IIa** - **IIa** |
| **JCS, 2013**  [12]  **About non-valvular atrial fibrillation** | “Aging is an independent major risk factor for embolism and anticoagulation is in principle required for elderly patients”. | **No grade** |
| **Ministry of health, Malaysia 2013**  [13]  **About pulmonary embolism** | “Anticoagulation in the elderly is similar to the general population but it must be noted that these patients may have underlying renal insufficiency”. | **B (evidence levels IIa, IIb, III)** |

| **Classes of recommendations** | **ACC/AHA guidelines** | **ESC guidelines** |
| --- | --- | --- |
| **COR I** | Benefit >>> risk   - Is recommended - Is indicated - Is beneficial | Evidence and/or general agreement that a given treatment is indicated, beneficial, useful, effective |
| **COR IIa** | Benefit >> risk (“routine practice”)   - Is reasonable - Can be useful, effective, beneficial | Conflicting evidence and/or a divergence of opinion about the usefulness/efficacy of the  given treatment: weight of evidence/opinion is in favor of usefulness/ efficacy   - Should be considered |
| **COR IIb** | Benefit ≥ risk (“case by case decision”)   - May/might be reasonable - May/might be considered - Usefulness/effectiveness is unknown/unclear/uncertain or not well established | Conflicting evidence and/or a divergence of opinion about the usefulness/efficacy of the given treatment: usefulness/ efficacy is less well established by evidence/ opinion   - May be considered |
| **COR III** | No benefit (benefit = risk)  Harm (risk > benefit)   - Is not recommended - Is not indicated, useful, effective, beneficial - Potentially harmful | Evidence or general agreement that the given treatment or procedure is not useful, effective, and in some cases may be harmful   - Is not recommended |

ACC . American College of Cardiology; AHA. American Heart Association; COR. Class of Recommendation; ESC . European Society of Cardiology.

*From: Capodanno D, Alfonso F, Levine GN, Valgimigli M, Angiolillo DJ. ACC/AHA Versus ESC Guidelines on dual antiplatelet therapy. J am Coll Cardiol. 2018;72: 2915–2931. doi:10.1016/j.jacc.2018.09.057*

**REFERENCES**

1. Joung B, Lee JM, Lee KH, Kim T-H, Choi E-K, Lim W-H, et al. 2018 Korean guideline of atrial fibrillation management. Korean Circ J. 2018;48: 1033–1080. doi:10.4070/kcj.2018.0339

2. Brieger D, Amerena J, Attia J, Bajorek B, Chan KH, Connell C, et al. National Heart Foundation of Australia and the Cardiac Society of Australia and New Zealand: Australian Clinical Guidelines for the diagnosis and management of atrial fibrillation 2018. Heart Lung Circ. 2018;27: 1209–1266. doi:10.1016/j.hlc.2018.06.1043

3. National Heart Association of Malaysia. Stable coronary artery disease 2018. Clinical Practice Guidelines. 2018. Available from: <https://www.malaysianheart.org/?p=cpg&a=1296>

4. Ibanez B, James S, Agewall S, Antunes MJ, Bucciarelli-Ducci C, Bueno H, et al. 2017 ESC Guidelines for the management of acute myocardial infarction in patients presenting with ST-segment elevation. Eur Heart J. 2017; 39(2):119-177. doi:10.1093/eurheartj/ehx393.

5. Baumgartner H, Falk V, Bax JJ, De Bonis M, Hamm C, Holm PJ, et al. 2017 ESC/EACTS Guidelines for the management of valvular heart disease. Eur Heart J. 2017;38(36):2739-2791. doi:10.1093/eurheartj/ehx391.

6. Roffi M, Patrono C, Collet JP, Mueller C, Valgimigli M, Andreotti F, et al. 2015 ESC Guidelines for the management of acute coronary syndromes in patients presenting without persistent ST-segment elevation: Task Force for the Management of Acute Coronary Syndromes in Patients Presenting without Persistent ST-Segment Elevation of the European Society of Cardiology (ESC). Eur Heart J. 2016;37(3):267–315. doi:10.1093/eurheartj/ehv320.

7. Kirchhof P, Benussi S, Kotecha D, Ahlsson A, Atar D, Casadei B, et al. 2016 ESC Guidelines for the management of atrial fibrillation developed in collaboration with EACTS. Eur Heart J. 2016;37(38):2893–962. doi:10.1093/eurheartj/ehw210.

8. Page RL, Joglar JA, Caldwell MA, Calkins H, Conti JB, Deal BJ, et al. 2015 ACC/AHA/HRS Guideline for the management of adult patients with supraventricular tachycardia: a report of the American College of Cardiology/American Heart Association Task Force on Clinical Practice Guidelines and the Heart Rhythm Society. J Am Coll Cardiol. 2016;67(13):e27–e115. doi:10.1016/j.jacc.2015.08.856.

9. Baker IDI Heart and Diabetes Institute. National evidence-based guideline on secondary prevention of cardiovascular disease in type 2 diabetes. 2015. Available from: http://t2dgr.bakeridi.edu.au

10. January CT, Wann LS, Alpert JS, Calkins H, Cigarroa JE, Cleveland JC Jr., et al. 2014 AHA/ACC/HRS guideline for the management of patients with atrial fibrillation. J Am Coll Cardiol. 2014;64(21):e1–e76. doi:10.1016/j.jacc.2014.03.022.

11. National Heart Association of Malaysia. Management of acute ST segment elevation myocardial infarction (STEMI) 2014 - (3RD Edition). Clinical Practice Guidelines. 2014. Available from: https://www.malaysianheart.org/?p=cpg&a=942.

12. JCS Joint Working Group. Guidelines for pharmacotherapy of atrial fibrillation (JCS 2013). Circ J 2014;78:1997-2021. doi:10.1253/circj.CJ-66-0092.

13. National Heart Association of Malaysia. Prevention and treatment of venous thromboembolism. Clinical Practice Guidelines. 2013. Available from : http://www.moh.gov.my/penerbitan/CPG2017/9005.pdf
